# Impact of a community-based pilot intervention to tackle childhood obesity: a ‘whole-system approach’ case study

**DOI:** 10.1186/s12889-020-09694-2

**Published:** 2020-11-30

**Authors:** E. W. Gadsby, S. Hotham, T. Eida, C. Lawrence, R. Merritt

**Affiliations:** 1 Centre for Health Services Studies, George Allen Wing, University of Kent, Canterbury, Kent CT2 7NF UK; 2 City of Westminster Council, Public Health Directorate, 64 Victoria Street, London, UK

**Keywords:** Childhood obesity, Prevention, Community-based, Health improvement, Whole systems, Evaluation, RE-AIM

## Abstract

**Background:**

Go-Golborne was a three-year pilot programme to test an innovative, community-based ‘whole system’ approach to preventing overweight in children in Golborne ward, London. Whilst there is a growing interest in local whole systems approaches to obesity, understandings of what they look like in practice are newly emerging. Go-Golborne was designed, implemented and evaluated within this context.

**Methods:**

The evaluation used a case-study design and theory of change approach to assess the effectiveness of the intervention. Height/weight measurements of children in the six participating primary schools were recorded annually for 4 years. For behavioural outcomes, children aged six-11 completed four annual on-line surveys (total 4331 responses). Parents were surveyed in year one and year four (177 responses). Three focus group discussions were held with children aged 10–11 (*N* = 21); interviews were conducted with parents (*N* = 11), and school representatives (*N* = 4). Stakeholders were surveyed twice (37 responses), and interviews were conducted with key stakeholders (*N* = 11). An extensive range of programme documents were reviewed and additional process data was collected from the programme team. The RE-AIM framework was used to synthesise findings and examine public health impact.

**Results:**

Go-Golborne reached a diverse range of partners across Golborne. Events were attended by over 3360 local children and families and all six primary schools in the ward actively engaged in activities. The proportion of children in the above healthy weight categories remained stable over time. A number of changes in home, school and neighbourhood environments to support healthy behaviour change were evidenced. There was some qualitative evidence of positive changes in children’s behaviours, though significant or sustained changes were not evidenced by the quantitative data.

**Conclusions:**

Go-Golborne helped stakeholders and parents to develop a shared commitment to improving healthy weight in children, to identify barriers to a healthy lifestyle, and to start to make changes in their services/behaviours. The campaigns and changes made at micro-level appeared to be insufficient, in the face of counteracting forces and personal factors, to achieve significant behaviour change within 3 years. This highlights the need for local initiatives to be reinforced by supporting action at regional, national and global levels.

**Supplementary information:**

**Supplementary information** accompanies this paper at 10.1186/s12889-020-09694-2.

## Background

The Go-Golborne intervention was developed by the Royal Borough of Kensington and Chelsea’s (RBKC) public health team to promote healthy lifestyles amongst children and families, as part of a broader programme to tackle childhood obesity. A third of year six children across RBKC are overweight or obese, and prevalence is above the London and national averages in several of the most deprived wards [1, 2]. Variations in prevalence are strongly linked to income and socio-economic status; higher rates of obesity tend to be concentrated in areas with high levels of deprivation. The RBKC council chose to pilot a targeted approach to identifying and addressing barriers to a healthy lifestyle at a community level within Golborne ward: an area with a diverse population and relatively high deprivation and obesity prevalence. There were around 900 children living in the ward, and over 1700 children attending six local schools [2].

There is a great deal of literature on behaviour modification or lifestyle change in the prevention and management of childhood obesity, influenced by several different theories, concepts and accounts of behaviour and behaviour change. Evidence of effectiveness for behavioural interventions has been mixed with small, short term effects on weight loss and Body Mass Index (BMI) [3, 4]. However research has highlighted key behaviour change techniques that are most likely to promote positive changes (e.g. provide information on the consequences of behaviour and environmental restructuring) [5]. Health behaviours are influenced by a range of socio-economic, cultural and environmental conditions, social and community networks and individual factors such as age and sex. Therefore, a combination of interventions that tackle population, community and individual-level factors are needed to help people change their behaviour in the longer term [6]. Systematic reviews of interventions and clinical guidelines indicate that successful interventions are complex and multi-component - aimed at changing both physical (or sedentary) activity and diet or healthy eating, and comprise multiple, potentially interacting methods of changing behaviour [3, 7–10]. In general, interventions which involve the whole community in complex interventions that target environments and upstream determinants appear to be more effective than those which simply target children [9].

The increased recognition of the complexity of obesity causation and prevention, and a frustration with the lack of success of efforts over the last few decades, has led to a growing interest in whole systems approaches (WSAs) [11, 12]. In theory, WSAs draw on understandings of complexity science and of complex adaptive systems that help to explain particular problematic situations and identify ways in which they might be improved. However, what is meant by a whole system is interpreted in different ways. In practice, they are often described in terms of ‘big picture’ thinking, where efforts are made to link together actions in a coordinated and integrated effort, across multiple sectors, to bring about change [13]. According to Public Health England, “a local whole systems approach responds to complexity through an ongoing, dynamic and flexible way of working … stakeholders agree actions and decide as a network how to work together in an integrated way to bring about sustainable, long-term systems change” (p.17) [12]. Whilst community-based and local WSAs to health and wellbeing are not the same thing, they share many common features, such as community engagement, long-term commitment, a focus on relationships and networks and dynamic understandings of causes and effects [14].

There is a paucity of evidence on the effectiveness of community-wide programmes displaying features of a WSA to prevent obesity. A systematic review of population-based whole-of-community obesity prevention interventions published in 2014 identified eight trials, none of which were undertaken in the UK [15]. The review suggested that such interventions can be effective in achieving *modest* reductions in population weight gain among children, but there is a paucity of evidence, particularly for the UK context. Since that review, there have been important additions to the evidence base, particularly from Australia where experiences in implementing community-based childhood obesity prevention projects in different contexts and communities found that the effectiveness of intervention strategies is dependent on individual and community factors. This reinforces the call for a systems approach whereby existing systems are modified [16]. The language, theory and practice of WSAs – certainly within the public health field - is still young. Understandings of how best to apply systems thinking and what a WSA to obesity looks like in practice are newly emerging [12] and there is little knowledge yet of what is most likely to work.

**Fig. 1:**
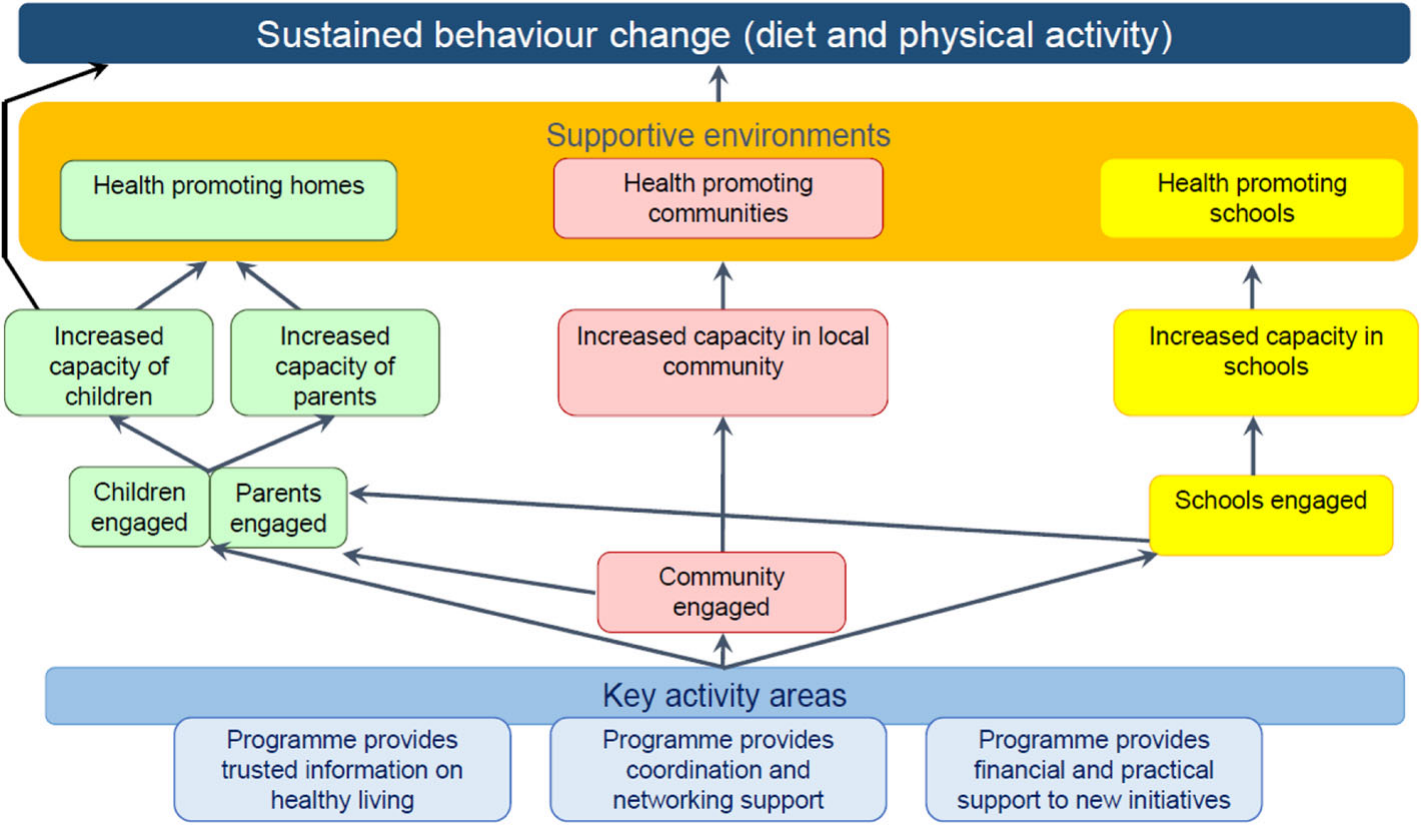
Theory of Change Diagram

Go-Golborne was designed in 2014 and implemented over 3 years (2015–2018). It sought to engage all those with a role in shaping the environments in which children live, learn and play in Golborne. The programme team described it as a WSA in that it combined “‘bottom-up’ community empowerment actions with ‘top-down’ interventions in a single initiative” and aimed to “use and optimize existing systems, build on local assets, connect multiple stakeholders, synchronize ongoing activities across multiple settings and stimulate further actions” (p.110) [17]. Its methodology and design are detailed in a separate paper [17], but it was informed by the best practice principles for community-based obesity prevention developed in Australia [18], the World Health Organisation Good Practice Appraisal Tool [19] and the EPODE approach to childhood obesity prevention [20]. The PESTEL framework (distinguishing political, economic, sociocultural, technological and physical and legal environments) was used to explore and describe the influences that hinder or support the adoption of healthy lifestyles in the community. From this, and through stakeholder engagement, a programme plan was developed that included: social marketing campaigns every 6 months, covering specific nutrition and physical activity themes; training and development opportunities for people working with children and families; the distribution and promotion of consistent messages on nutrition and physical activity; environmental improvements, working with council departments, local agencies and other stakeholders such as local retailers; and community development activities, including a grant scheme for each theme, local events and other ad hoc support. The high-level programme theory was that by engaging the whole community and stakeholders within the ward and across the council in a geographically-focused initiative, locally appropriate and co-developed activities would be designed and delivered to raise awareness and understanding of the issues, and encourage and support behaviour change amongst children and their families (see Theory of Change, Fig. 1).

Researchers at the University of Kent were commissioned to design and conduct a robust research evaluation (from May 2015 to April 2019). This paper provides a summary of the overarching findings. A thorough analysis of the quantitative data, and a more detailed report of the process evaluation findings will be available in separate articles (in progress).

## Methods

**Fig. 2:**
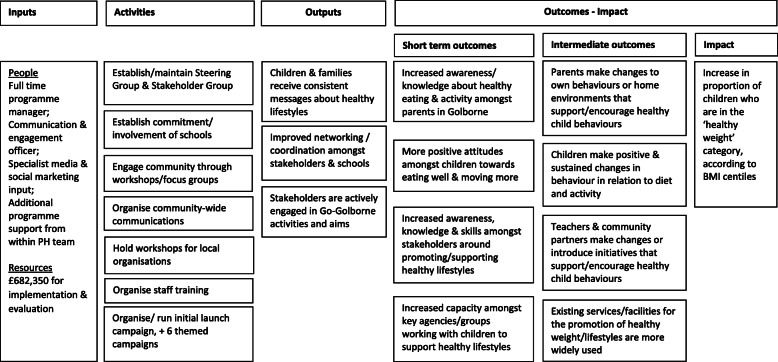
Logic Model of Go Golborne Programme

The evaluation was designed to answer questions associated with process, outcomes, and implications for future programmes and policy. It took a theory of change approach [23–24], which clarified the programme’s aims, objectives and outcomes and articulated the assumptions underlying the programme’s design (see Logic Model, Fig. 2). Data collection, management and analysis was guided by the RE-AIM framework [25], which focuses on essential programme elements (reach, efficacy, adoption, implementation, and maintenance) that can improve the sustainable adoption and implementation of evidence-based interventions.

Data was collected and analysed to measure programme reach, assess implementation fidelity, and examine programme context, from: eight steering group meetings; ten stakeholder group meetings; event log forms (completed by the programme team); progress reports; eight newsletters; three in-depth interviews with the programme co-ordinator; and attendance records and other programme documentation.

A non-experimental case study design and mixed methods were used to evaluate a range of indicators, in accordance with the logic model, at baseline, mid-term and follow-up where possible. Data sources included:

- Height/weight measurements of all children in six primary schools each year from 2016 to 2019 (collected by the community health trust as an extension to the existing National Child Measurement Programme (NCMP));
- Child questionnaires (on-line, self-complete in classroom – see Additional files 1 and 2) with children in years two to six in the six schools: January–March each year from 2016 to 2019 (total responses = 4331);
- Parent questionnaires (self-complete on paper or on-line – see Additional file 3): early 2016 and early 2019 with parents of children in six primary schools (total responses = 177);
- Partner questionnaires (self-complete on-line – see Additional file 4): mid-2016 and mid-2018, with partner organisations (total responses = 37);
- Semi-structured interviews with school representatives: May–June 2018 (*N* = 4);
- Semi-structured interviews with key stakeholders (representing various sectors): 2017 (*N* = 8) and 2018 (*N* = 3);
- Focus group discussions with purposive sample of year six children: 2018 (*N* = 21, in three focus groups);
- Semi-structured interviews with parents: 2018 (*N* = 6); and focus group with mothers at local children’s centre (*N* = 5);
- Other documentary information from the programme team.

All interviews were either face-to-face or via telephone. Most were audio-recorded with consent from participants; for two parent interviews, detailed notes were taken. Focus group discussions were audio-recorded, except the one with mothers at a children’s centre, for which detailed notes were taken by a scribe.

A thorough review of all existing validated questionnaires identified none that would meet our objectives and be appropriate for primary school-aged children. For example, when exploring how to capture active play, few of the existing questionnaires dealt with physical activity or exercise that might be considered ‘active play’ (e.g. Physical Activity Questionnaire for Older Children). The Day in the Life Questionnaire (DILQ) [26] asked what children did yesterday at morning break, lunchtime, and after school, but there was no attempt to measure frequency, intensity or duration. All questionnaires therefore were collaboratively designed by the evaluation and programme teams. Subject experts were consulted, and the survey structure and some specific questions were drawn from our review of existing validated questionnaires. The child questionnaire was designed to measure any significant change, over time, in the population health behaviours of children in Golborne. Questions were in a simple and suitable format (adapting those used in existing questionnaires for young children such as the DILQ), with embedded audio files and clear graphics to aid comprehension. Children in years 5 and 6 were asked 14 additional questions taken directly from the Child Nutrition Questionnaire [27] to assess attitudes towards eating fruit and vegetables. The questionnaire was pre-tested with seven children aged seven to 11 in order to explore comprehension, retrieval/recall, judgement and response. It was then piloted in a primary school with similar pupil profile (*N* = 91) and subsequently refined by the evaluation and programme teams.

The parent/carer questionnaire collected additional information on children’s behaviours, assessed parent awareness and knowledge around key themes, and assessed parents’ behaviours in relation to supporting healthy eating/activity in their children. The partner questionnaire explored the support partners received from the Go-Golborne team, the extent of partner engagement, how information was being received and used, whether capacity had been strengthened, and whether community partners were doing anything new or different to support children in making positive behaviour changes.

All data sources were analysed separately according to their methodological requirements. Survey data were analysed using statistical analysis (SPSS version 25). Outliers (which varied according to survey question and ranged from 24 to 115 participants) were removed and descriptive statistics computed. For data generated from the Child Nutrition Questionnaire, answers were provided on a five-point scale from 1 ‘strongly agree’ to 5 ‘strongly disagree’, with higher scores indicating a more negative attitude towards fruit and vegetables, and lower scores indicating a more positive attitude. Example questions include: ‘Eating vegetables makes me feel healthy’ and ‘I like the taste of most fruit’. Cronbach’s alpha for subscales on attitudes to fruit and vegetables indicated good reliability (fruit α = .81 veg, α = .94). A Linear Mixed-Effects Model (LMM) analysis was conducted to explore potential differences in mean scores. The standard level of significance (*p* < .05) was used to examine patterns in the data from 2016 to 2019.

Height and weight data for school years one to five were combined with the routine NCMP dataset to add in Reception and year six. For the extended NCMP data, the LMSgrowth tool was used to calculate BMI, BMI Standard Deviation (z-score) and BMI percentile based on sex, date of birth, date of measurement and height and weight values [28]. Weight classifications were determined using the UK90 BMI reference curves [29]. For routine NCMP the validated percentiles as provided by Public Health England were used (LMS results in the same BMI groups for these measures). For clinical BMI groups the following centiles were used as cut-offs: underweight: ≤2.3, healthy weight: 2.4 to < 90.9, overweight 90.9 and over, very overweight 97.7 and over. The analysis consisted of a series of pupil counts under different variables, e.g. by BMI classification.

Qualitative data was analysed using thematic analysis of either full audio transcripts or detailed notes, using the theory of change as an analytical framework, to which sub-themes were added inductively [30]. Two researchers independently coded a sub-set of transcripts until agreement and confidence was reached. One researcher conducted the remaining coding, bringing any arising issues to the research team for discussion and consensus. Analysis templates were populated in Microsoft Word for each data source to identify the key data organised into themes; data within each theme were synthesised into thematic statements. This enabled a close link to the data to be maintained to ensure analysis remained grounded in the data, and to ensure that a range of data sources, contributed to building explanatory models. For the overarching analysis across data sets, prominent and recurring themes from across the data were extracted, matched and cross-compared to develop an explanatory case for the propositions at the heart of the Go-Golborne programme [31]. Rival explanations were also sought and interrogated.

## Results

This section presents a summary of the main evaluation findings in relation to the elements in the RE-AIM framework.

### Reach

The Go-Golborne events were attended by over 3360 local children and families, with the most popular events attracting more than 1000 participants. Given the estimate of 900 children living in the ward, and over 1700 children attending the six primary schools working with the project, this represents excellent reach into the community. Stakeholders praised the diverse range of partners that reflected the local community and offered greater relevance and reach. The involvement of all six primary schools enabled access to a large number of local children and families. Children and parents also engaged with Go-Golborne at after-school clubs and holiday activities. Parents with pre-school children had less contact with the programme content, though some recognised the logo through posters in the Children’s Centre or park events.

### Efficacy

Qualitative data suggested that children’s knowledge about healthy foods improved over the course of the programme, and they now had an improved capability to make small changes in their dietary choices, where supported. Stakeholders had noticed positive changes in knowledge/awareness amongst children, particularly related to certain Go-Golborne campaigns.

*“I think it's had a really positive impact on the community; … children are more aware of their healthy eating choices, they are aware of what they should eat and shouldn't eat”.* (Statutory partner, interviewed 2017)

*“My children… they love all the projects and they came home and kept talking about it and my son was like, 'oh mummy I'm not having a doughnut, because it contains so much sugar!'”.* (Parent, interviewed 2017)

Key messages around physical activity do not appear to have been absorbed so readily by the children. There was a greater sense of decisions being outside of the children’s control:

*“… sometimes there’s good stuff going on but then if you are busy or like I have younger brothers then you can’t always go”* (Child in Focus Group Discussion, 2018).

Parents reported that Go-Golborne had raised awareness of healthy eating and activity in a fun and enjoyable way, and had provided them with greater motivation to further support healthy choices for their children. The follow-up parent questionnaires, however, did not suggest an improvement in knowledge around key health-related recommendations.

Data from partners, parents, teachers and children appeared to suggest that attitudes amongst children and parents were shifting. Quantitative data gathered via the Child Nutrition Questionnaire (CNQ) (for years 5–6, *N* = 1692) identified a positive shift in attitudes (i.e. lower value scores on CNQ) towards eating fruit and vegetables across the 4 year period. The relationship between cohort and attitudes towards vegetables showed significant variance in intercepts across participants, var.(*u*_0*j*_) = 2.65, *X*^2^ (9) = 130.18, *p* < .01. Results from the LMM suggest that attitudes in 2019 (M = 6.56, SD = 3.70) towards vegetables improved compared to at the start of Go-Golborne in 2016 (M = 15.17, SD = 3.58), *F*(3,778.77) = 236.14, *p* < .01, (CI 95% = 4.89, 5.83).

The relationship between cohort and attitudes towards fruit also showed significant variance in intercepts across participants, var.(*u*_0*j*_) = 3.54, *X*^2^ (9) = 184.12, *p* < .01. Results from the LMM suggest that attitudes in 2019 (M = 6.64, SD = 3.08) towards fruit improved compared to levels in 2016 (M = 17.53, SD = 3.08), *F*(3,721.16) = 1201.94, *p* < .01, (CI 95% = 9.95, 10.76).

The child questionnaire did not collect information on attitudes towards physical activity (due to the need to keep the length manageable), but rather focused on measuring changes in behaviour. Qualitative data highlighted that children associated physical activity with having fun and socialising with friends, rather than ‘being healthy’. However, having fun and socialising was also closely linked to the use of electronic devices. Other children, who appeared to enjoy more physical activity, pointed to the barriers to taking part and the lack of opportunities, both in school and out.

Partners reported that their collaboration with Go-Golborne improved their reach into schools or community settings, increased the creativity and relevance of the messages they delivered, and linked the campaign messages to their own frameworks. They reported making many useful new contacts, and benefiting from participating in Go-Golborne events through an increased awareness of local services. Responses to the stakeholder questionnaire highlighted, for example, new collaborations between different organisations and groups. Training provided by the programme enabled local staff members to feel more confident in delivering consistent messages about health and weight when working with families. Most partners felt the programme improved their ability to support healthy lifestyles in the community, e.g. through developing new skills or knowledge around supporting children and families.

A large proportion of parents responding to the 2019 questionnaire reported making positive changes to improve their children’s diet, increase the amount of physical activity, and decrease the amount of screen time their children engaged in. For example: 49% of parents responding to the survey reported making changes to reduce sugar (with cutting down on sweets and/or sugary snacks and having smaller portions of sugary foods/drinks being the most frequently cited examples), 56% to reduce salty/fatty snacks and 60% to increase fruit and vegetable consumption; 46% of parents reported making changes to be more active in travel to/from school; and 50% of parents reported making changes to reduce screen time. Partners and teachers reported seeing some of these changes beginning to happen, although they highlighted that there was still much progress to be made, that some families needed more support than others, and that there was a need to keep the momentum going.

Schools and local community venues/services were starting to make positive changes to support healthier diets and activity. Many different examples of changes were mentioned by organisations, including swapping the snacks and drinks provided for healthier alternatives, promoting healthier vending machines, organising and promoting walks and bike rides, creating and promoting new ways of encouraging active play, and running non-screen sessions during holiday times. Children, parents and partners referred to the changes that they had seen in local shops and venues, with, for example, some noticing a shift towards healthier options being available in shops and greater visibility of fruit and vegetables at street level. Teachers also detailed the continued and additional ways in which they were making healthier choices easier in school by, for example, having easy access to drinking water, offering active after school clubs, and proving fruit/vegetable snacks to key stage two pupils. These positive changes were being noticed by parents, with the majority of those responding to the 2019 survey agreeing that their child’s school actively supports healthy eating and active movement.

In the second stakeholder questionnaire, partners described a higher uptake of local activities – both those facilitated by their own organisation and those in other settings (e.g. local leisure centres), and there were increased referrals to child healthy weight services.

Across the six behaviour change themes, there was little quantitative evidence from the surveys of positive, sustained shifts in children’s behaviours. Most behaviours fluctuated across the four cohorts. The parent questionnaires also confirmed that there was much progress to be made in improving children’s behaviours to meet recommended levels. For example, in 2019, 65% of responding parents thought their child ate fewer than the recommended 5 a day; only 16% of parents said their child took part in vigorous activity on 5 days or more; and 27% of parents reported that their youngest child engages in two or more hours of screen time on a typical school day (60% on a typical weekend day). The behaviour change data is reported in full elsewhere (in progress).

However, qualitative data suggested some positive shifts in behaviours. For example, partners reported that parents no longer brought sweet snacks or drinks to the activity sessions; and local shops and businesses reported fewer children buying sweets where partners had banned unhealthy snacks.

The data collected on children’s heights and weights indicated that the proportion of children in the ‘healthy weight’ category (according to BMI centiles) remained stable, with no statistically significant change over the four-year time period. The proportion of children in the ‘overweight’ and ‘very overweight’ categories also remained stable over time.

### Adoption

The Go-Golborne partnership comprised 110 organisations and businesses, including schools, nurseries, community centres, mosques, market traders and corner shops. A small core of partners (six to nine organisation representatives, including a local councillor) met as the Steering Committee eight times during the programme. A larger stakeholder group, averaging 25 attendees, met ten times during the programme. In total, over 100 stakeholder partners representing at least 62 organisations attended at least once. Organisations included those from the third sector, Council departments, health and leisure partners and others, which brought a diversity of local knowledge, contacts and expertise to the table. Partners were also engaged through training sessions, small grants delivery, use and dissemination of resources, and in the planning and delivery of events. Between six and 25 agencies were involved in each of the community events. Further details of key programme activities and their uptake are provided in Table 1. This highlights a high level of adoption within the community by a wide range of partners who interact with children and families.

**Table 1:** Description of Go Golborne community activities involving local partners

Activity	Details	Uptake
Stakeholder meetings	10 quarterly stakeholder meetings to plan, reflect, evaluate and report work, and invite input from partners.	Over 100 partners attended from over 62 organisations.
Training for partners	Six tailored training sessions delivered on: healthy eating and nutrition; physical activity and play; nutritional guidelines and cooking on a budget; active health and delivering physical activity; sugar smart ‘train the trainer’; being a walk leader.	Between eight and 22 participants attended from a range of organisations. At least 75 partners took part overall.
Events	Seven community events aligned with the campaigns with a fun community focus.	An average of: 26 partners involved in event planning; 19 involved in delivery.
Campaign resources	Go Golborne created 46 different project resources and distributed 76,000 items to children and families.	All partners received the resources for information and distribution. Eight out of 10 partner survey respondents said that they distributed resources to their service users.
Campaign Grants	Grants of up to £2000 available to local partners to deliver community activities and/or for organisational development.	52 grants were distributed to local partners, including primary schools. Four out of 10 partner survey respondents had applied for and received a grant.
Work with primary schools	Delivery of healthy lifestyle messages (e.g. through facilitated assemblies and sharing of resources) and support to strengthen school practices and policies	All six local primary schools engaged; four schools received a total of seven grants. Five achieved Healthy Schools awards and two worked with MyTime Active who delivered activity workshops for students.
Strengthening the food environment	Local partners and businesses supported to develop healthy policies and practice through campaigns and partnership.	40 partners made pledges to be more Sugar Smart; 10 pledged to make organisational or policy changes. 77 businesses achieved a Healthier Catering Commitment award. Environmental Health piloted additional sugar-smart criteria with seven businesses as part of their Heathy Catering award scheme.
Extension projects (commissioned work)	Shop Healthy Golborne: Rice Marketing worked with local traders to audit, position and promote healthier products	Three local convenience stores participated. They introduced 77 new healthier lines and actively promoted them.
	Fit for Kids: Health Education Partnership developed a kitemark system for community organisations to develop best practice in promoting healthy lifestyles.	Three organisations piloted the programme. One achieved the award, others provided valuable feedback to improve the tool. Two more organisations were subsequently working towards the award.

### Implementation

The six themed community-wide social marketing campaigns formed the backbone of Go-Golborne’s multi-strategy approach. Around this backbone, implementation was flexible to adapt to changing circumstances and to lessons learned. This adaptability proved to be of crucial importance: first, when due to cut-backs 5 months in, programme staffing was significantly reduced (the full-time communication and engagement officer was cut to minimal communications support), and second, when in June 2017, the Golborne community was rocked by the tragic fire at the neighbouring Grenfell Tower. The event and its aftermath traumatised members of the local community, stretched local services, took a great deal of focus and attention, and damaged relationships, particularly between the community and the Council. The Go-Golborne staff were extremely sensitive to this context. Despite some inevitable implications for programme delivery, all the campaigns largely ran as anticipated. Information was disseminated via 76,000 original health promotion resources; the majority of partners found the information to be highly trustworthy, relevant and useful. The seven community events were widely supported by partners and attended by the local community. The campaigns generated positive messages to which stakeholders and community members responded well. Training was delivered to over 75 local staff/volunteers, with consistently high feedback. Many opportunities were provided for network-building and partnership development. Twenty-six partners received 52 Go-Golborne grants to deliver activities related to the campaigns, and four schools used grants for theme-based activities. Stakeholders reported that Go-Golborne was responsive to local concerns, and aligned itself with existing/similar services and programmes, reducing the potential for overlap or unnecessary additional work, and helping to ensure that involvement was a positive experience.

### Maintenance

Relationships forged in the early days of the programme were actively maintained throughout. The staggered delivery of the campaigns helped to ensure that partners could be engaged in each different theme, helping to keep their interest in the programme overall. Existing infrastructures were built on for programme delivery and programme actions were integrated into the practice of partner organisations. The collaborative way in which campaign messages and resources were designed and delivered helped to ensure that they became embedded within the minds of many key change agents (such as those working with children). Partners explained that the programme resources will continue to be used and the knowledge and connections made during the programme will continue to be valuable. The emphasis on simple messages, and realistic, achievable ideas increased the likelihood that elements of the programme would be embedded into routine practice. As these partners commented:

*“The culture of our organisation is starting to shift slowly towards understanding and enabling healthier choices”* (Local voluntary sector partner, partner survey 2016)

*“We’re going to maintain these things; we’re not going to change anything. I took all [the Go Golborne] banners, and we’ve got the Unplug and Play poster out in the playground as a constant reminder and [the programme has] left a legacy because we have all these great things in place. So like with me, I campaign for public health, for children’s health, so it will always be on the top of my agenda when it comes to outcomes for children – it will always stay, it’s fixed”* (Go-Golborne Partner, interviewed 2018).

*“I think it’s sustainable because it’s not too straining on the schools to keep doing it … I think that it’s a good thing to do”* (Teacher, interviewed 2018)*.*

As shown in Table 1, many activities, particularly around improving the food environment, prompted partners to implement changes in routine organisational practices and policies.

## Discussion

Go-Golborne was developed as a pilot approach to identifying and addressing barriers to a healthy lifestyle at community level, and a potentially effective way of reducing child obesity. The challenge set by the council was ambitious. It aimed, within 3 years, to engage the whole community and stakeholders within the ward and across the council, to design and deliver locally appropriate and co-developed activities to raise awareness and understanding of the issues, and encourage and support behaviour change amongst children and their families. The scale and complexity of this challenge, and the importance of context in shaping the success of the programme, was acknowledged early on by the programme and evaluation teams.

The design and conduct of the evaluation was fraught with challenges that are now comprehensively discussed in the literature [21, 23, 32–35]. In particular, these were multiple programme components, action at multiple levels, the importance of context, the flexible and evolving nature of the programme, the breadth and long-term nature of the outcomes being pursued, and the absence of appropriate control groups for comparison purposes [36, 37]. The case study design sought to take account of these challenges by taking a theory-of-change approach with mixed methods and a strong process evaluation. It enabled an in-depth empirical investigation of the situation to understand the how and why questions within the evaluation. However, the evaluation was inherently political [38] and there were expectations to manage about what the evaluation would and wouldn’t be able to ‘prove’. The case study design enabled the integration of qualitative and quantitative data from a variety of sources to give an in-depth analysis of the situation and the context. Whilst qualitative data was inevitably from a small sample, efforts were made to reduce response bias, particularly by using purposive sampling methods and pro-active approaches (e.g. working with community groups to help engage specific participants) to ensure we incorporated perspectives of diverse audiences. The evaluation provided a detailed picture of programme operations and resulted in a rich understanding of how and why programme operations related to outcomes. However, in the measuring of behaviour-related outcomes, the team were obliged for pragmatic reasons to rely on self-reported data, which has obvious limitations in terms of recall and social desirability bias. Moreover, since there were no existing tools that suited our purpose given the need to assess change across six behaviour change themes, the evaluation used bespoke (and therefore unvalidated) questionnaires. Low sample sizes from the parent surveys limited our ability to detect small intervention impacts and generalise the findings to the whole community. Another limitation is sampling bias (participation was voluntary). Behaviour data reported by young children should be treated with particular caution. Evaluation findings should be considered, therefore, with these caveats in mind.

In Golborne, throughout the programme period, data collected via child surveys indicated that children (at a population level) did not make significant changes to their eating and physical activity behaviours. This is perhaps unsurprising given the time-frame, the scale of the programme, the complexity of the issue, and the limitations in the evaluation methodology. Similar findings, demonstrating inconsistent and limited success in changing healthy eating and physical activity-related behaviours, have been found in the evaluation of other comparable interventions [16]. As with the experience in Australia [16], aspects of the local context were important in relation to the achievement of behaviour change objectives. In particular, many factors outside of the programme’s sphere of control, such as the relative poverty of many Golborne residents and the Grenfell fire, affected both implementation and context.

Although population behaviours did not appear to change significantly, there was some evidence that a supportive environment was starting to be developed in Golborne’s homes, schools and neighbourhoods. This was created by giving community stakeholders information, skills and motivation to support children in making healthy choices. The programme sought to raise awareness and knowledge of healthy eating and physical activity through social marketing campaigns, at the same time as making micro-environmental changes through informing, engaging and supporting a range of stakeholders. The locally-designed campaigns were developed with strong community involvement and used established social structures of the community. Existing evidence suggests that such approaches are more likely to be implemented and sustained [39, 40]. However, evaluation data suggested that the raised awareness and knowledge brought about by the campaigns and the changes made at micro-level were not sufficient to achieve a reduction in child overweight. This is consistent with other literature that highlights the importance of being realistic about the potential of such programmes to alter the outcomes of a system as complex and extensive as that driving the weight status of populations, especially within a three-year period [41, 42].

Go-Golborne demonstrated that it *is* possible to bring stakeholders together to develop a shared commitment to tackling overweight, to recognise the part they can play, and to start to make changes in their services/behaviours. There are strengths in this design that relate to the principles of practice for collective impact, which has proven to be a powerful approach in tackling a wide range of issues in communities all over the world [43]. The findings demonstrate that capacity to tackle overweight within the Golborne community was strengthened in a number of ways, including knowledge, skills, resources and opportunities. This is significant as existing evidence suggests that a community-wide, capacity-building approach to reducing child obesity is flexible, cost-effective, sustainable, equitable and safe, and has the potential to influence the underlying social and economic determinants of health [44]. Go-Golborne strengthened and leveraged the interaction of human capital, organisational resources and social capital to help tackle child overweight as a collective problem. Most importantly, it did this in a way that strengthened community identity, built frameworks to facilitate sustainable change, and empowered the community through a strength-based approach and inclusive practice. In the context of the tragic fire at Grenfell Tower, a wide range of issues related to inequity and mental health came to the fore which couldn’t help but affect neighbouring Golborne. In this context, Go-Golborne not only managed to maintain its momentum throughout the period, but also demonstrated the value of its approach in terms of building trust, strengthening networks and reinforcing a community identity.

Go-Golborne aimed to take a ‘whole systems’ approach at a local level, which is consistent with a growing body of evidence and current thinking around how best to tackle obesity. A significant four-year action research project carried out during the same period as Go-Golborne resulted in a ‘whole systems approach to obesity’ guide and resources, published in July 2019 [12]. Using the guide as a framework of best practice, Go-Golborne could be considered as having done a good job of implementing a ‘whole systems approach’ to obesity, albeit at a very local (ward) level. It secured senior-level support and established the necessary governance and resource structure to implement the approach; it built a compelling narrative and a shared understanding of why obesity matters locally and how it can be addressed; it brought stakeholders together to understand the local system and agree a shared vision; it oversaw a number of collaborative and aligned actions; it maintained momentum by developing a stakeholder network; and it critically reflected on its approach and considered opportunities for strengthening the process. However, the explicit use of systems tools (like causal loop diagrams or group based modelling, for example) did not feature in attempts to map the local system. In addition, as a local community-centred project within a large borough, Go-Golborne placed more emphasis on Golborne-based actors and actions than on change within and driven by the Council. For improved impact, the Council should seek to scale up this systems approach to working across the whole borough – preferably in concert with a similar London-wide and indeed UK government-wide whole systems approach.

## Conclusion

Go-Golborne represents an important attempt to implement an evidence-informed, community-based, WSA to childhood obesity prevention in a deprived inner-city ward, within a local government context that is experiencing some of the tightest financial restrictions in recent history [45].

The findings from the evaluation of the Go-Golborne intervention demonstrate that this kind of approach can establish firm foundations for supporting healthier diet and physical activity related behaviours amongst children, through engaging children and their families, schools, and the wider community. The intervention helped stakeholders and parents to develop a shared commitment to tackling overweight, to identify barriers to a healthy lifestyle, and to start to make changes in their services/behaviours. Key to this engagement was running a positive, fun and locally-tailored campaign with excellent reach into the community, broad adoption by partners, and flexible implementation plans that took account of the local context and adapted to changes and challenges. These foundations were deemed to be crucial for building trust (and therefore for acceptability of the intervention), and for maintaining the programme’s momentum in the longer-term. However, the findings also highlight the complexity of and time taken to significantly alter population behaviours, and consequently weight status. The campaigns and changes made at micro-level appeared to be not sufficient, in the face of counteracting forces and personal factors, to achieve significant behaviour change within 3 years. This highlights first, the need for local initiatives to be reinforced by supporting action at regional, national and global levels, and second, the need for all initiatives to be seen as part of a longer term vision for childhood obesity prevention.

## Supplementary information

**Additional file 1.** The survey distributed to children in years 2 to 4. (PDF 1269 kb)

**Additional file 2.** The survey distributed to children in years 5 to 6. (PDF 1282 kb)

**Additional file 3.** The survey distributed to parents/carers in 2019. (PDF 872 kb)

**Additional file 4.** The survey distributed to Go-Golborne partners in 2018. (PDF 350 kb)

## Data Availability

The data that support the findings of this study are available from Royal Borough of Kensington and Chelsea Council (RBKC) Public Health Team, but restrictions apply to the availability of these data, which were used under license for the current study, and so are not publicly available. Data are however available from the authors upon reasonable request and with permission of RBKC.

## Abbreviations

RBKC: Royal Borough of Kensington and Chelsea
BMI: Body Mass Index
WSA: Whole Systems Approach
NCMP: National Child Measurement Programme
DILQ: The Day In the Life Questionnaire
LMM: Linear Mixed-Effects Model
CNQ: Child Nutrition Questionnaire
