# Supplementary material for: Impact of a community-based pilot intervention to tackle childhood obesity: a ‘whole-system approach’ case study
Source: BMC Public Health. 2020 Nov 30;20:1818. doi: 10.1186/s12889-020-09694-2 (PMC7708136; doi:10.1186/s12889-020-09694-2)
Supplement: Supplementary file 1 — Additional file 1. The survey distributed to children in years 2 to 4. (PDF 1269 kb) [file 12889_2020_9694_MOESM1_ESM.pdf]

Go Golborne Pupil Questionnaire for years 2, 3 and 4

Click the play button below each question to hear the questions read aloud as below:

Press next to continue

First please tell us a bit about yourself

1. Your teacher will give you a four digit number. Please write your **own** four digit number in the box below.

2. How old are you?

- ○ 6 years
- ○ 7 years
- ○ 8 years
- ○ 9 years

3. Are you a boy or a girl?

Boy	Girl
○	○

4. Which primary school do you go to?

- ○ Barlby Primary School
- ○ Bevington Primary School
- ○ St Charles R C Primary School
- ○ St Mary's Catholic Primary School
- ○ St Thomas CE Primary School
- ○ Ark Brunel

Next we are going to ask you some questions about what you do in the morning before school

5. Where did you have breakfast **this morning**?

- At home

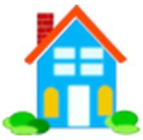

- At school

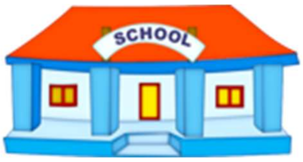

- On the way to school

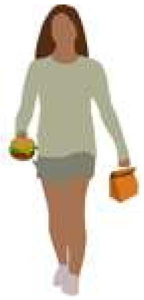

- I didn't have any breakfast
- Somewhere else (please tell us):

6. Did you eat **fruit** with breakfast **today**?

- Yes
- No

What did you have?

Banana

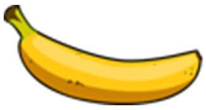

Fruit Salad

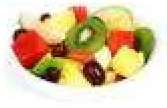

Orange or clementin e

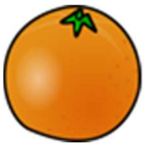

☐ Berries
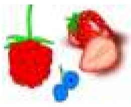

☐ Apple
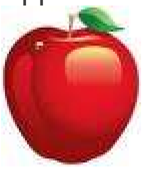

☐ Pear
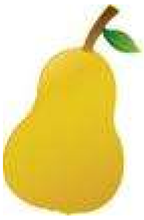

☐ Cherry
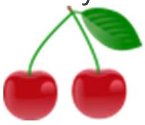

☐ Melon
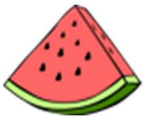

☐ Grapes
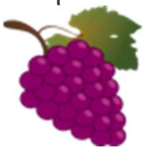

☐ Kiwi fruit
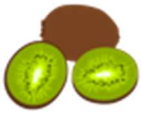

☐ Another fruit (please tell us)
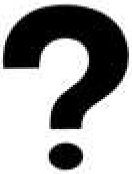
[ ]

7. Did you eat **vegetables** with breakfast **today**?

○ Yes
○ No

What did you have?

☐ Carrot
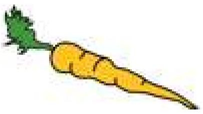

☐ Cauliflower
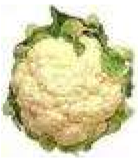

☐ Baked beans
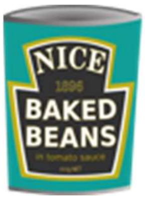

☐ Cabbage
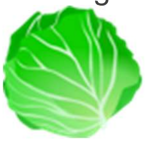

☐ Salad
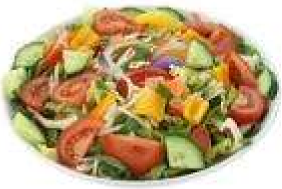

☐ Mushrooms
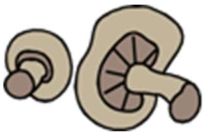

- [ ] Broccoli

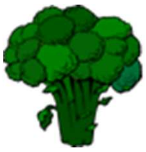

- [ ] Peas or green beans

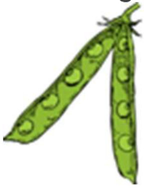

- [ ] Sweetcorn

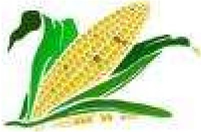

- [ ] Another vegetable (please tell us)

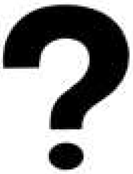

______

8. On school days, how often do you usually watch TV or play on the computer **before school**?

Never (0 days)	Not very often (1 or 2 days)	Most days (3 or 4 days)	Every day (5 days)
○	○	○	

9. How do you **usually** travel to school in the morning?

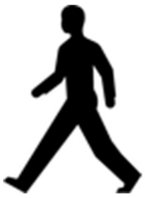

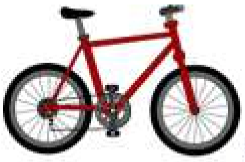

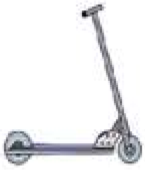

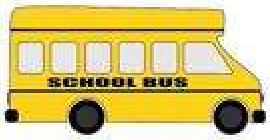

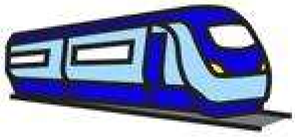

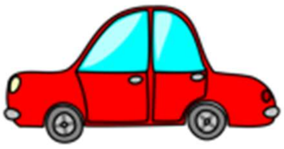

Walk or Run	Cycle or Scoot	By Bus, train or tube	By Car
○	○	○	○

10. Did you have a snack or drink on the way to school **this morning**?

- [ ] Crisps

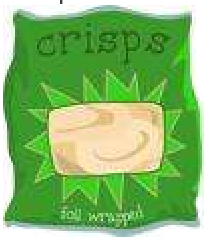

- [ ] Rice cake or crackers

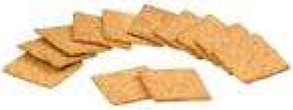

- [ ] A juice or juice drink

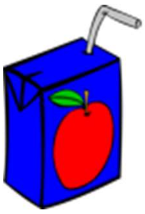

Biscuit, cake or cereal bar

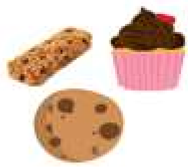

Burger, hot dog, pizza, chips or fried chicken

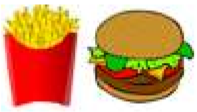

Milkshake

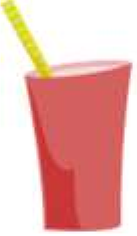

Sweets or Chocolate

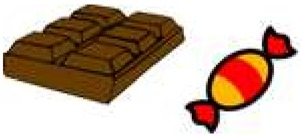

A regular fizzy drink

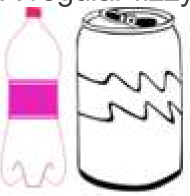

Milk (not flavoured)

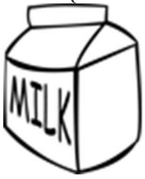

Fruit or Veg

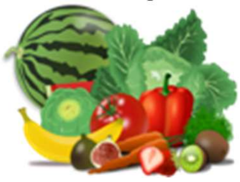

A diet drink

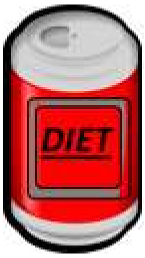

Water

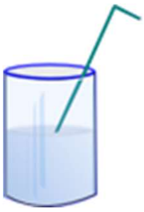

I didn’t have anything to eat or drink on the way to school

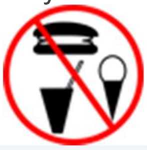

Something else (please tell us)

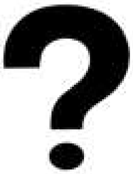

Now for some questions about the school day

11. What do you **mostly** do at **morning playtime**?

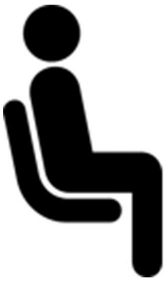

Sit around

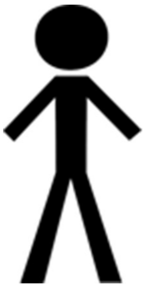

Stand around

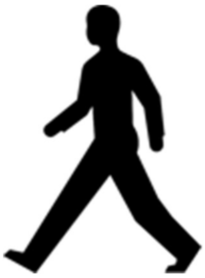

Walk around

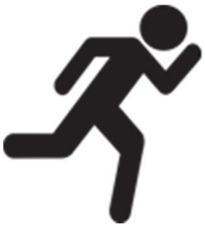

Run around

12. What do you **mostly** do at lunch time? Do not include the time you spend eating lunch.

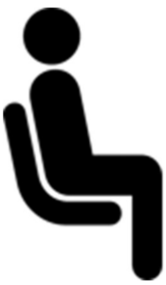
Sit around

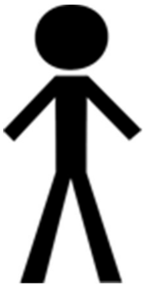
Stand around

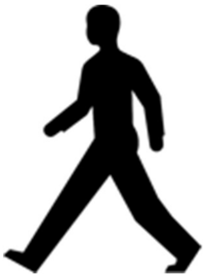
Walk around

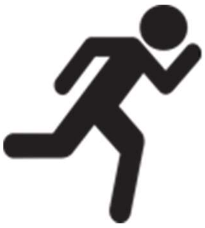
Run around

13. Where do you usually get your lunch from?

- Packed Lunch

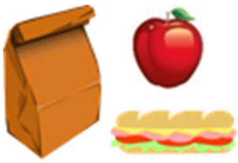

- School Dinner

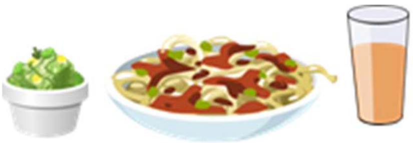

- No lunch
- Somewhere else (please tell us):

14. Did you eat any **fruit** with lunch **yesterday**?

- Yes
- No

What did you have?

- Banana
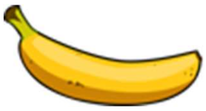

- Fruit Salad
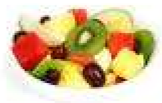

- Orange or clementine
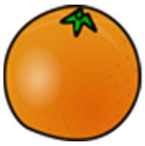

Berries
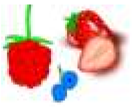

Apple
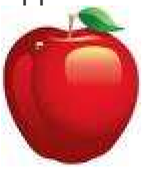

Pear
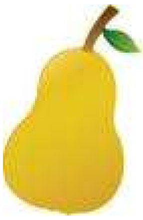

Cherry
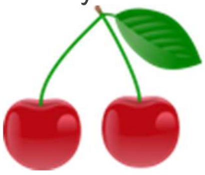

Melon
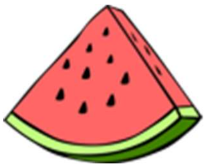

Grapes
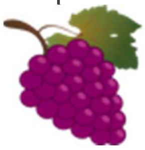

Kiwi fruit
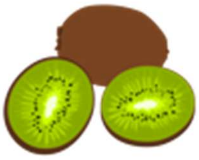

Another fruit (please tell us)
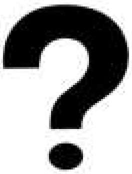

15. Did you eat any **vegetables** with lunch **yesterday**?

- Yes
- No

What did you have?

Carrot
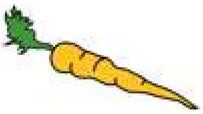

Cauliflower
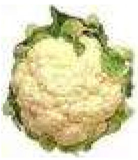

Baked beans
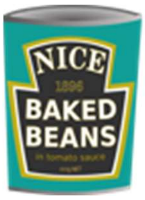

Cabbage
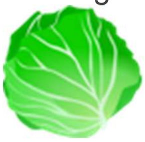

Salad
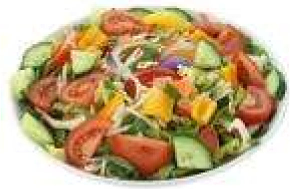

Mushrooms
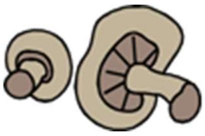

Broccoli
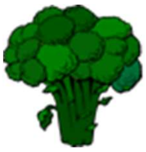

Peas or green beans
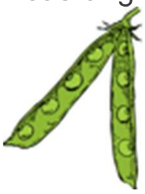

Sweetcorn
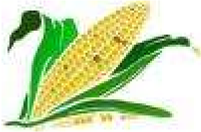

☐ Another vegetable (please tell us)

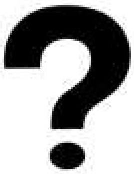

Now we would like to know what you did <u>on the way home from school or after school club</u>

16. How do you **usually** travel home after school?

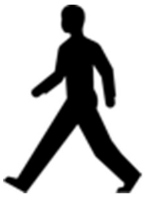
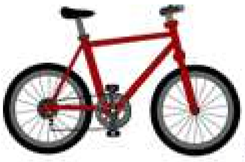
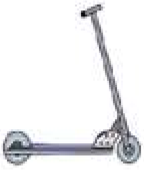
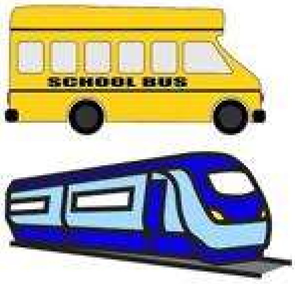

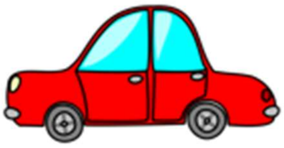

Walk or Run	Cycle or Scoot	By bus, train or tube	By Car
○	○	○	○

17. Did you have a snack or drink **<u>on the way home from school</u>** yesterday?

☐ Crisps

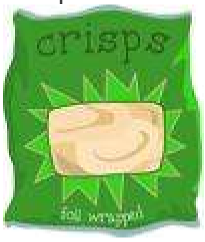

☐ Rice cake or crackers

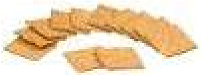

☐ A juice or juice drink

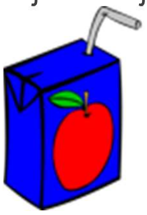

☐ Biscuit, cake or cereal bar

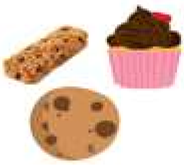

☐ Burger, hot dog, pizza, chips or fried chicken

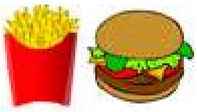

☐ Milkshake

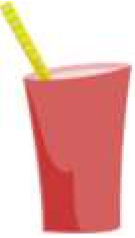

☐ Sweets or Chocolate

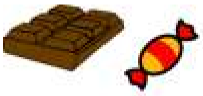

☐ A regular fizzy drink

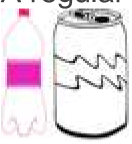

☐ Milk (not flavoured)

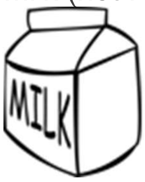

Fruit or Veg

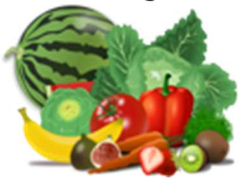

A diet drink

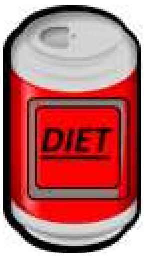

Water

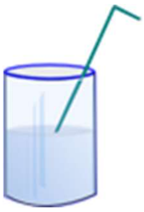

I didn't have anything to eat or drink on the way home

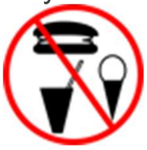

Something else (please tell us)

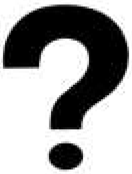

Next we would like to know what you did <u>outside school time</u>

18. On any day **last week**, did you take part in any individual or team sports, or any other physical activities? Which of these activities did you do?

Swimming

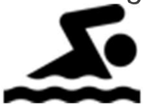

Cycling

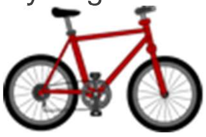

Skating or skateboarding

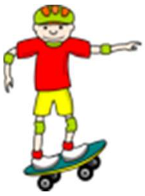

Ball games

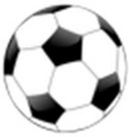

Racket or Stick sports

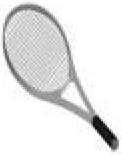

Dance

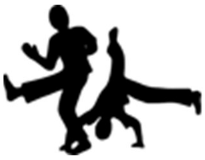

Athletics

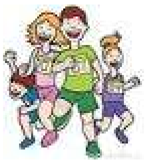

Skippng

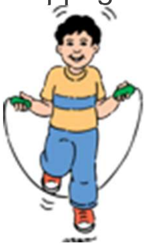

Martial Arts

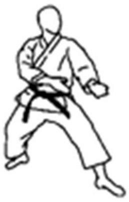

Gymnastics
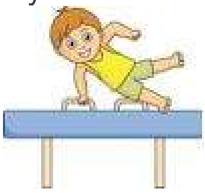

I do something else [please tell us]:
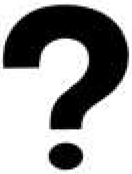

I didn't take part in any physical activities last week
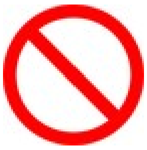

19. How often do you **usually** run around or play in the garden, playground or park **after school**?

Never (0 days)	Not very often (1 or 2 days)	Most days (3 or 4 days)	Every day (5 days)
◯	◯	◯	◯

20. How often do you **usually** watch TV or play on the computer **after school, but before your evening meal**?

Never (0 days)	Not very often (1 or 2 days)	Most days (3 or 4 days)	Every day (5 days)
◯	◯	◯	

21. Did you have a snack or drink <u>after you got home and **before your evening meal**</u> **yesterday**?

Crisps
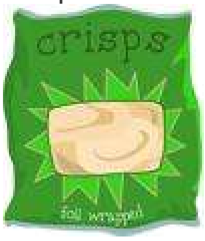

Rice cake or crackers
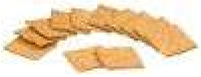

A juice or juice drink
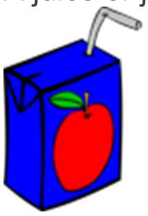

Biscuit, cake or cereal bar
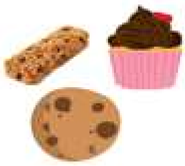

Burger, hot dog, pizza, chips or fried chicken
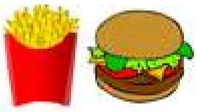

Milkshake
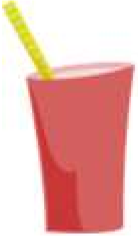

Sweets or Chocolate
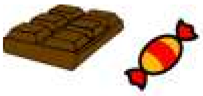

A regular fizzy drink
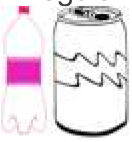

Milk (not flavoured)
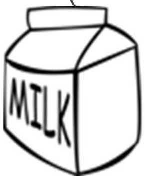

Fruit or Veg

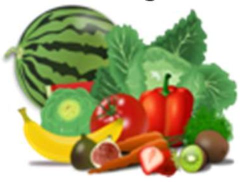

A diet drink

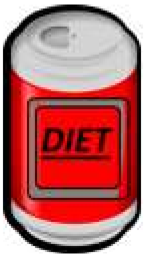

Water

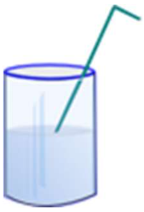

I didn’t have anything to eat or drink

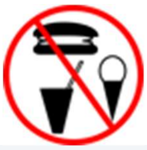

Something else (please tell us)

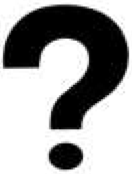

Now for some questions about your evening meal

22. How often do you usually have your evening meal in a restaurant, cafe or from a takeaway shop?

Once a month or less

2-3 times a month

About once a week

More than once a week

23. Did you eat **vegetables** with your evening meal **yesterday**?

- Yes
- No

What did you have?

Carrot

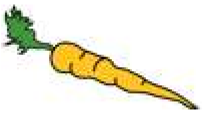

Cauliflower

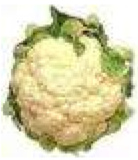

Baked beans

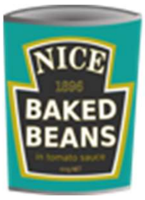

Cabbage

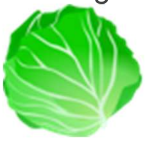

Salad

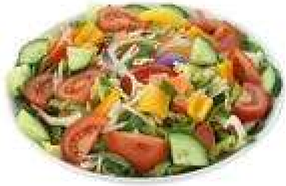

Mushrooms

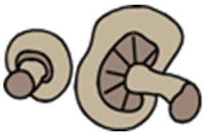

☐ Broccoli
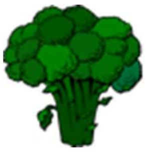

☐ Peas or Green beans
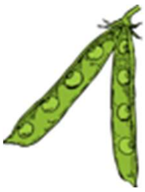

☐ Sweetcorn
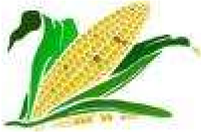

☐ Another vegetable (please tell us)

?

24. Did you eat **fruit** as pudding/dessert yesterday?

- ○ Yes
- ○ No

What did you have?

☐ Banana
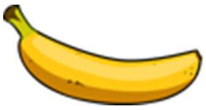

☐ Fruit Salad
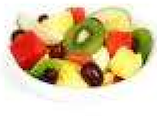

☐ Orange or clementine
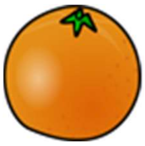

☐ Berries
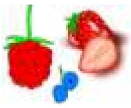

☐ Apple
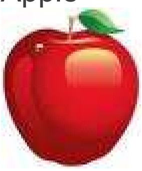

☐ Pear
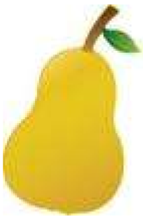

☐ Cherry
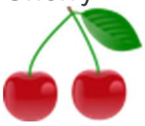

☐ Melon
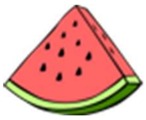

☐ Grapes
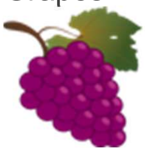

☐ Kiwi fruit
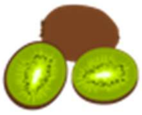

☐ Another fruit (please tell us)

?

26. On a school day, how often do you usually watch TV or play on the computer in the evening, **<u>after your evening meal</u>**?

Never (0 days)	Not very often (1 or 2 days)	Most days (3 or 4 days)	Every day (5 days)
○	○	○	

27. Did you have a snack or drink **<u>after your evening meal and before you went to bed</u> last night**? Do not include your pudding/dessert.

☐ Crisps

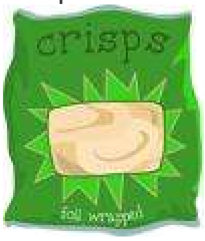

☐ Rice cake or crackers

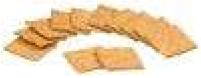

☐ A juice or juice drink

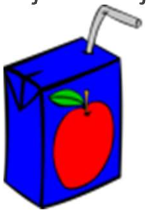

☐ Biscuit, cake or cereal bar

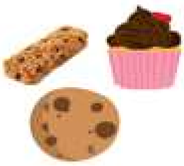

☐ Burger, hot dog, pizza, chips or fried chicken

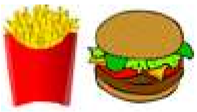

☐ Milkshake

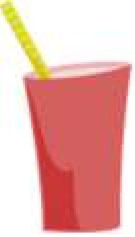

☐ Sweets or Chocolate

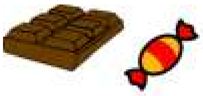

☐ A regular fizzy drink

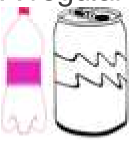

☐ Milk (not flavoured)

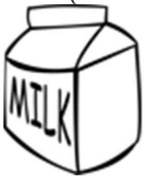

☐ Fruit or Veg

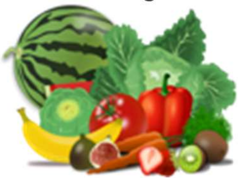

☐ A diet drink

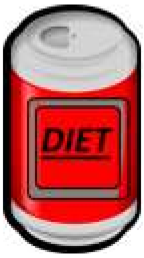

☐ Water

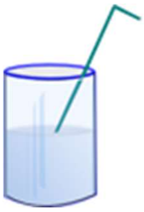

I didn’t have anything to eat or drink after dinner

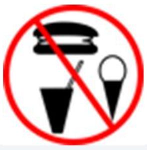

Something else (please tell us)

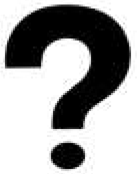

Well done - you've reached the end of the survey!

Please click 'next' to finish ->

Thank you very much :)
