# Supplementary material for: Impact of a community-based pilot intervention to tackle childhood obesity: a ‘whole-system approach’ case study
Source: BMC Public Health. 2020 Nov 30;20:1818. doi: 10.1186/s12889-020-09694-2 (PMC7708136; doi:10.1186/s12889-020-09694-2)
Supplement: Supplementary file 2 — Additional file 2. The survey distributed to children in years 5 to 6. (PDF 1282 kb) [file 12889_2020_9694_MOESM2_ESM.pdf]

THE ROYAL BOROUGH OF KENSINGTON AND CHELSEA

CHSS University of Kent Centre for Health Services Studies

Go Golborne Pupil Questionnaire for years 5 and 6

Click the play button below each question to hear the questions read aloud as below:

Press next to continue

First please tell us a bit about yourself

1. Your teacher will give you a four digit number. Please write your **own** four digit number in the box below.

2. How old are you?

- 9 years
- 10 years
- 11 years

3. Are you a boy or a girl?

Boy | Girl

4. Which primary school do you go to?

- Barlby Primary School
- Bevington Primary School
- St Charles R C Primary School
- St Mary's Catholic Primary School
- St Thomas CE Primary School
- Ark Brunel

Next we are going to ask you some questions about what you do in the morning before school

5. Where did you have breakfast **this morning**?

- At home

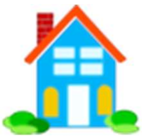

- At school

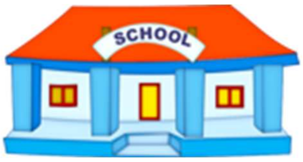

- On the way to school

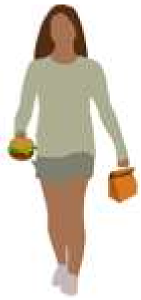

- I didn't have any breakfast
- Somewhere else (please tell us):

6. Did you eat **fruit** with breakfast **today**?

- Yes
- No

What did you have?

Banana

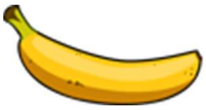

Fruit Salad

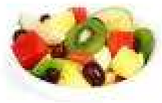

Orange or clementin e

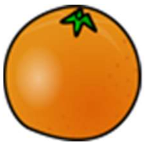

Berries

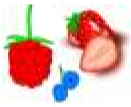

Apple

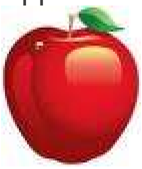

Pear

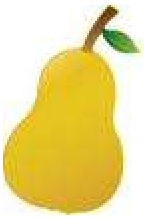

Cherry

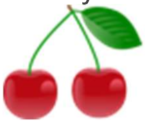

Melon

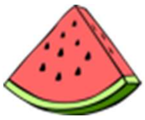

Grapes

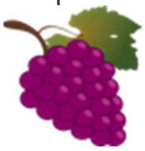

Kiwi fruit

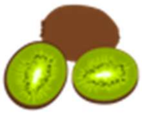

Another fruit (please tell us)

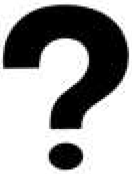

7. Did you eat **vegetables** with breakfast **today**?

- Yes
- No

What did you have?

Carrot

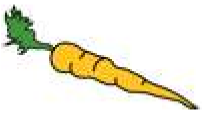

Cauliflower

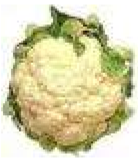

Baked beans

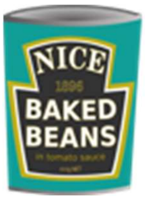

Cabbage

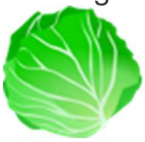

Salad

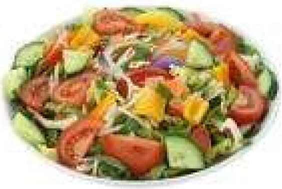

Mushrooms

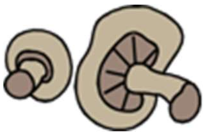

Broccoli

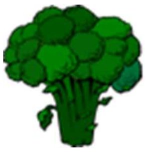

Peas or green beans

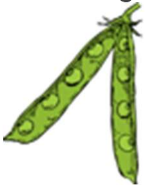

Sweetcorn

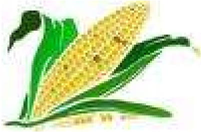

Another vegetable (please tell us)

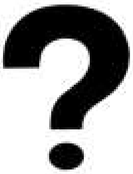

8. On school days, how often do you usually watch TV or play on the computer **before school**?

Never (0 days)	Not very often (1 or 2 days)	Most days (3 or 4 days)	Every day (5 days)

9. How do you **usually** travel to school in the morning?

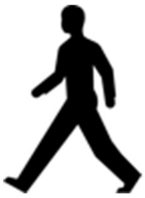

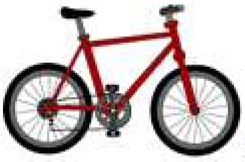

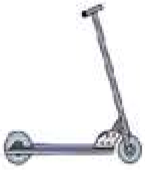

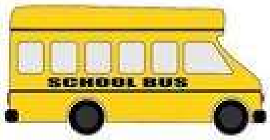

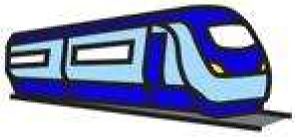

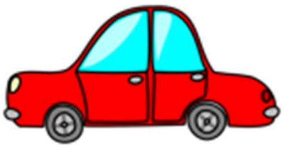

Walk or Run	Cycle or Scoot	By Bus, train or tube	By Car

10. Did you have a snack or drink on the way to school **this morning**?

Crisps

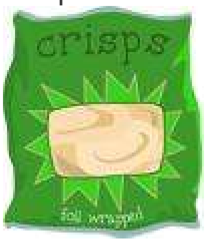

Rice cake or crackers

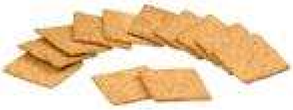

A juice or juice drink

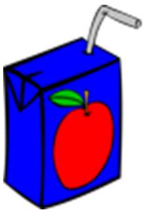

☐ Biscuit, cake or cereal bar

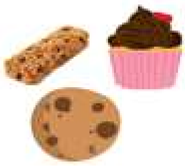

☐ Burger, hot dog, pizza, chips or fried chicken

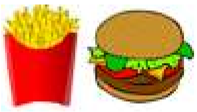

☐ Milkshake

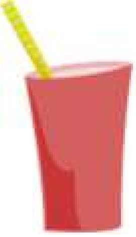

☐ Sweets or Chocolate

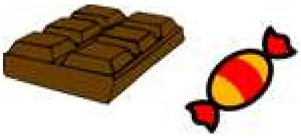

☐ A regular fizzy drink

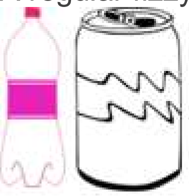

☐ Milk (not flavoured)

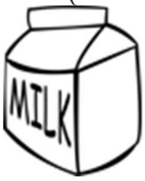

☐ Fruit or Veg

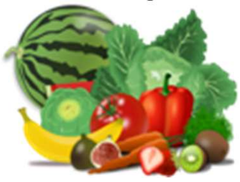

☐ A diet drink

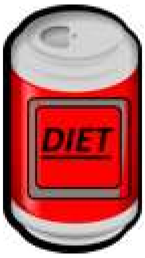

☐ Water

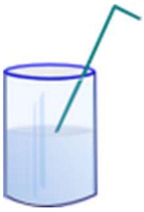

☐ I didn’t have anything to eat or drink on the way to school

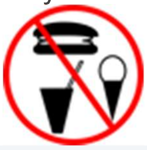

☐ Something else (please tell us)

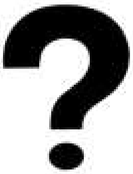

______

Now for some questions about the school day

11. What do you **mostly** do at **morning playtime**?

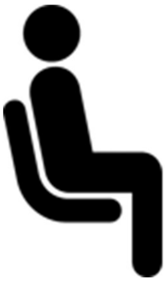

Sit around ○

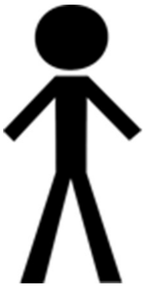

Stand around ○

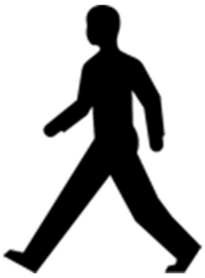

Walk around ○

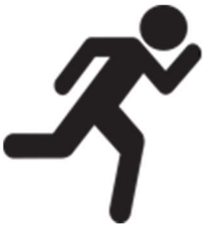

Run around

12. What do you **mostly** do at lunch time? Do not include the time you spend eating lunch.

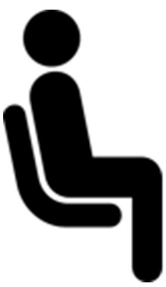
Sit around

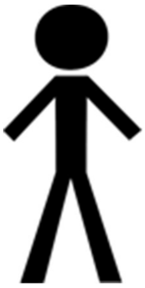
Stand around

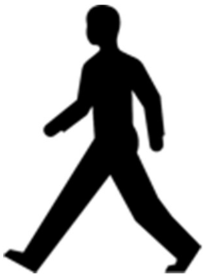
Walk around

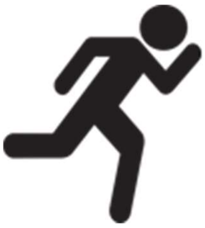
Run around

13. Where do you usually get your lunch from?

Packed Lunch

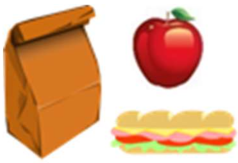

School Dinner

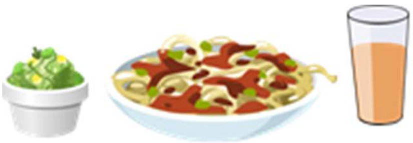

No lunch

Somewhere else (please tell us):

14. Did you eat any **fruit** with lunch **yesterday**?

Yes

No

What did you have?

Banana

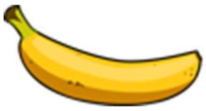

Fruit Salad

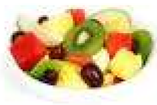

Orange or clementine

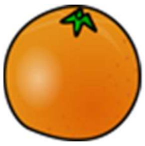

☐ Berries
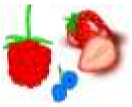

☐ Apple
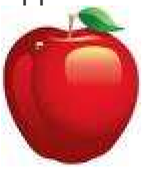

☐ Pear
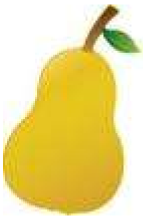

☐ Cherry
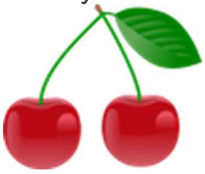

☐ Melon
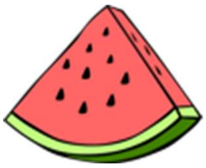

☐ Grapes
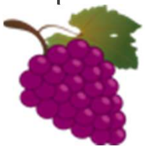

☐ Kiwi fruit
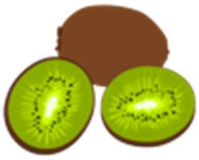

☐ Another fruit (please tell us)
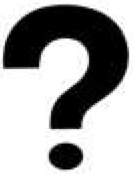

____

15. Did you eat any **vegetables** with lunch **yesterday**?

○ Yes
○ No

What did you have?

☐ Carrot
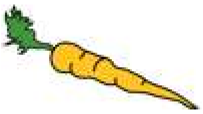

☐ Cauliflower
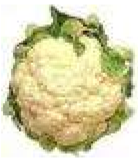

☐ Baked beans
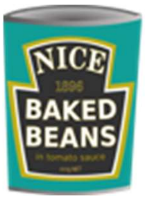

☐ Cabbage
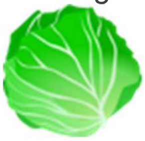

☐ Salad
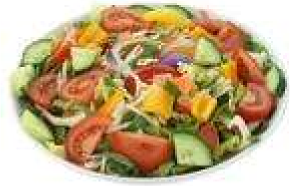

☐ Mushrooms
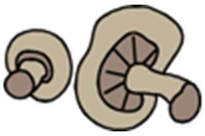

☐ Broccoli
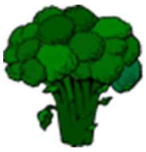

☐ Peas or green beans
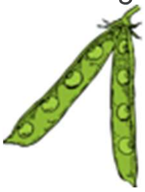

☐ Sweetcorn
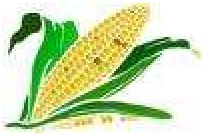

☐ Another vegetable (please tell us)

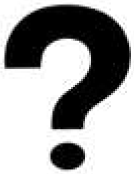

Now we would like to know what you did <u>on the way home from school or after school club</u>

16. How do you **usually** travel home after school?

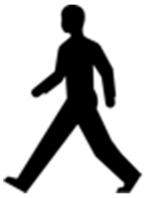
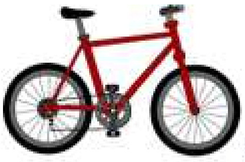
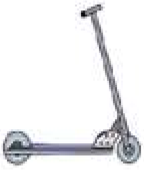
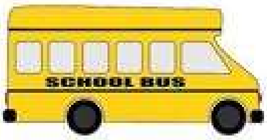

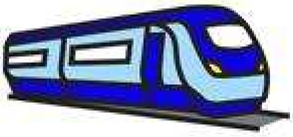
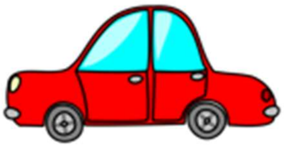

Walk or Run	Cycle or Scoot	By bus, train or tube	By Car
○	○	○	○

17. Did you have a snack or drink **<u>on the way home from school</u>** yesterday?

☐ Crisps

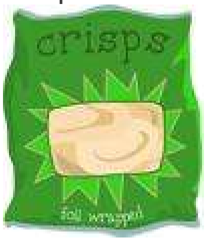

☐ Rice cake or crackers

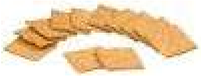

☐ A juice or juice drink

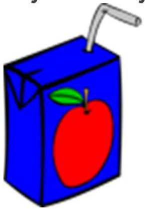

☐ Biscuit, cake or cereal bar

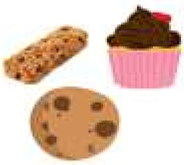

☐ Burger, hot dog, pizza, chips or fried chicken

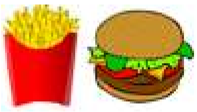

☐ Milkshake

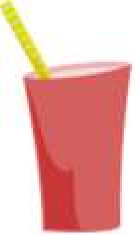

☐ Sweets or Chocolate

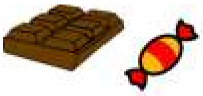

☐ A regular fizzy drink

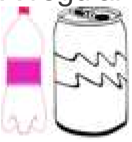

☐ Milk (not flavoured)

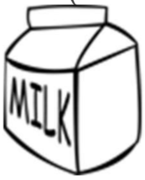

☐ Fruit or Veg

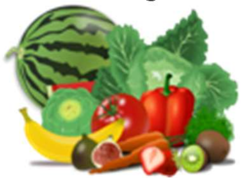

☐ A diet drink

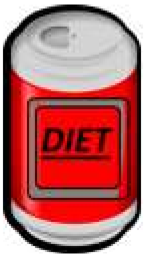

☐ Water

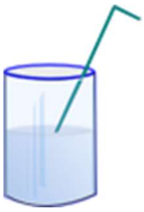

☐ I didn't have anything to eat or drink on the way home

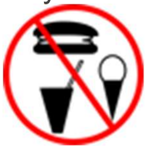

☐ Something else (please tell us)

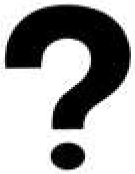

Next we would like to know what you did <u>outside school time</u>

18. On any day **last week**, did you take part in any individual or team sports, or any other physical activities? Which of these activities did you do?

☐ Swimming

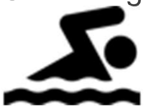

☐ Cycling

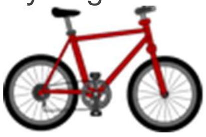

☐ Skating or skateboarding

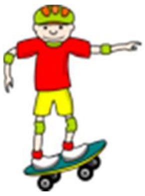

☐ Ball games

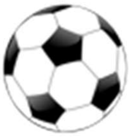

☐ Racket or Stick sports

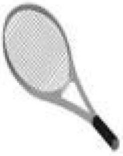

☐ Dance

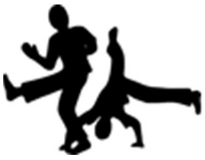

☐ Athletics

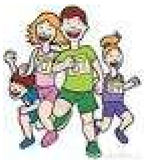

☐ Skippng

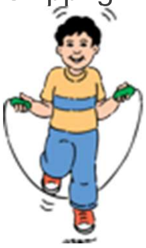

☐ Martial Arts

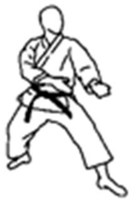

☐ Gymnastics

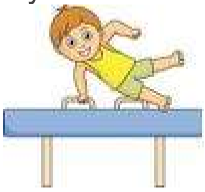

☐ I do something else [please tell us]:

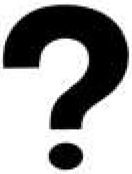

☐ I didn't take part in any physical activities last week

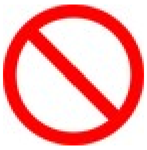

19. How often do you **usually** run around or play in the garden, playground or park **after school**?

Never (0 days)	Not very often (1 or 2 days)	Most days (3 or 4 days)	Every day (5 days)
○	○	○	○

20. How often do you **usually** watch TV or play on the computer **after school, but before your evening meal**?

Never (0 days)	Not very often (1 or 2 days)	Most days (3 or 4 days)	Every day (5 days)
○	○	○	

21. Did you have a snack or drink <u>after you got home and **before your evening meal**</u> **yesterday**?

☐ Crisps

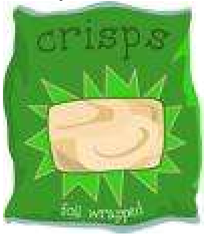

☐ Rice cake or crackers

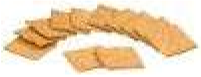

☐ A juice or juice drink

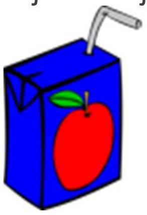

☐ Biscuit, cake or cereal bar

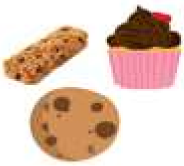

☐ Burger, hot dog, pizza, chips or fried chicken

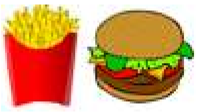

☐ Milkshake

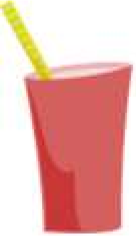

☐ Sweets or Chocolate

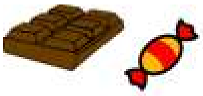

☐ A regular fizzy drink

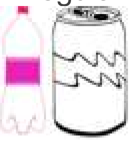

☐ Milk (not flavoured)

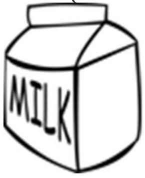

☐ Fruit or Veg
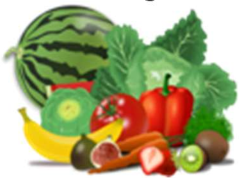

☐ A diet drink
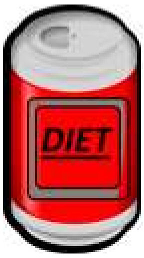

☐ Water
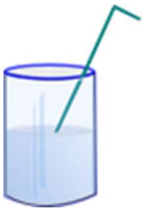

☐ I didn't have anything to eat or drink
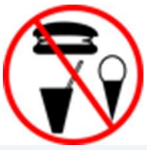

☐ Something else (please tell us)
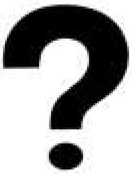

Now for some questions about your evening meal

22. How often do you usually have your evening meal in a restaurant, cafe or from a takeaway shop?

Once a month or less	2-3 times a month	About once a week	More than once a week
○	○	○	

23. Did you eat **vegetables** with your evening meal **yesterday**?

- ○ Yes
- ○ No

What did you have?

☐ Carrot
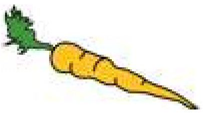

☐ Cauliflower
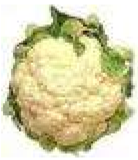

☐ Baked beans
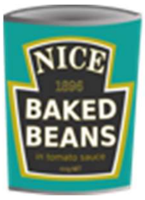

☐ Cabbage
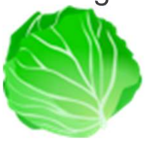

☐ Salad
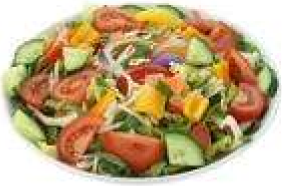

☐ Mushrooms
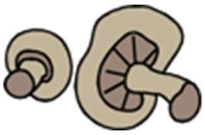

☐ Broccoli
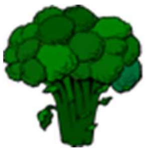

☐ Peas or Green beans
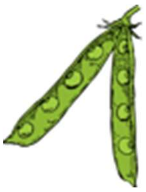

☐ Sweetcorn
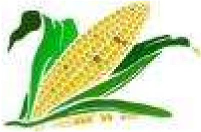

☐ Another vegetable (please tell us)
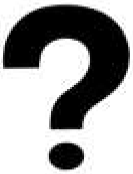

24. Did you eat **fruit** as pudding/dessert yesterday?

- ○ Yes
- ○ No

What did you have?

☐ Banana
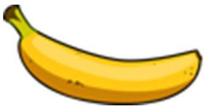

☐ Fruit Salad
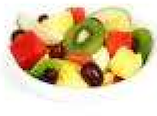

☐ Orange or clementine
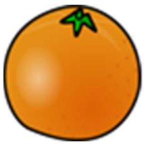

☐ Berries
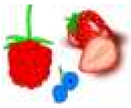

☐ Apple
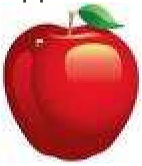

☐ Pear
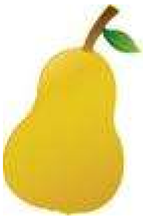

☐ Cherry
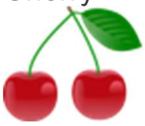

☐ Melon
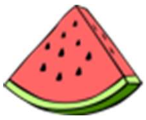

☐ Grapes
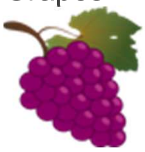

☐ Kiwi fruit
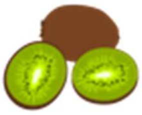

☐ Another fruit (please tell us)
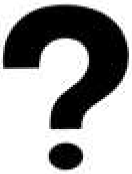

26. On a school day, how often do you usually watch TV or play on the computer in the evening, **after your evening meal**?

Never (0 days)	Not very often (1 or 2 days)	Most days (3 or 4 days)	Every day (5 days)
○	○	○	

27. Did you have a snack or drink **after your evening meal and before you went to bed last night**? Do not include your pudding/dessert.

☐ Crisps

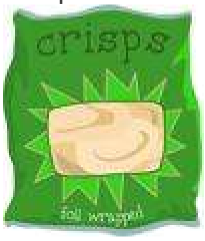

☐ Rice cake or crackers

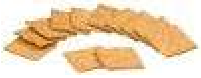

☐ A juice or juice drink

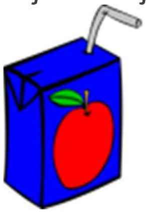

☐ Biscuit, cake or cereal bar

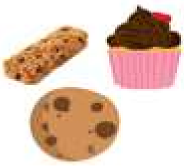

☐ Burger, hot dog, pizza, chips or fried chicken

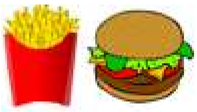

☐ Milkshake

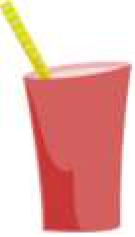

☐ Sweets or Chocolate

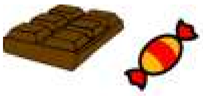

☐ A regular fizzy drink

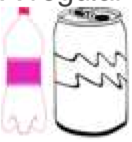

☐ Milk (not flavoured)

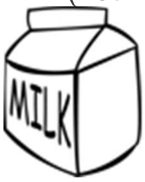

☐ Fruit or Veg

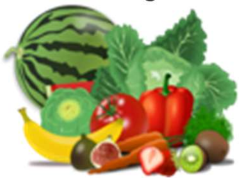

☐ A diet drink

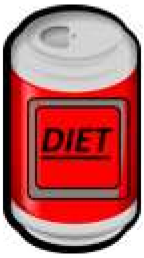

☐ Water

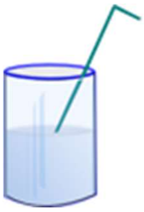

☐ I didn’t have anything to eat or drink after dinner

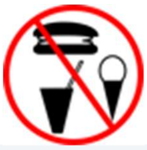

☐ Something else (please tell us)

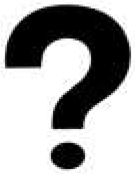

Finally, we would like to know what you think

28. How strongly do you agree or disagree with the following statements?

	Strongly agree	Agree	Not sure	**Dis**agree	Strongly **Dis**agree
Eating vegetables makes me feel healthy	○	○	○	○	○
I like the taste of many vegetables	○	○	○	○	○
In my home, vegetables are served at dinner most nights	○	○	○	○	○
I like tasting new vegetables that I haven't tried before	○	○	○	○	○
It is easy to prepare vegetables to eat, e.g make a salad	○	○	○	○	○
Eating fruit makes me feel healthy	○	○	○	○	○
I like the taste of most fruit	○	○	○	○	○
Fruit is an easy snack	○	○	○	○	○
I like tasting new fruits that I haven't tried before	○	○	○	○	○
In my home fruit is available to eat at any time	○	○	○	○	○
I like to drink water	○	○	○	○	○
I ask my parents to buy food or drink that	○	○	○	○	○

	Strongly agree	Agree	Not sure	**Dis**agree	Strongly **Dis**agree
I see advertised on television					
My parents encourage me to eat fruit and vegetables	○	○	○	○	○
Most of my teachers encourage the students to eat fruit and vegetables	○	○	○	○	○

29. Last question now! Please tell us what you think about the Go Golborne project.

Do you agree, are not sure, or disagree with these statements?

	I agree :)	I'm not sure :/	I disagree :(
I know more about healthy food and drink choices now	○	○	○
I don't like Go Golborne telling me what to do	○	○	○
It's quite easy to make healthy changes to what I eat and drink, and how much I move about	○	○	○
I try to move more now than I used to	○	○	○

Well done - you've reached the end of the survey!

Please click 'next' to finish ->

Thank you very much :)
