# Supplementary material for: Impact of a community-based pilot intervention to tackle childhood obesity: a ‘whole-system approach’ case study
Source: BMC Public Health. 2020 Nov 30;20:1818. doi: 10.1186/s12889-020-09694-2 (PMC7708136; doi:10.1186/s12889-020-09694-2)
Supplement: Supplementary file 3 — Additional file 3. The survey distributed to parents/carers in 2019. (PDF 872 kb) [file 12889_2020_9694_MOESM3_ESM.pdf]

THE ROYAL BOROUGH OF KENSINGTON AND CHELSEA

CHSS University of Kent Centre for Health Services Studies

A Questionnaire for Parents or Carers of Primary School Children in Golborne / W10

Section 1 – About where you live

1. How healthy do you think the Golborne/W10 area is for children to grow up in?

- ○ Very unhealthy
- ○ Fairly unhealthy
- ○ Neither healthy nor unhealthy
- ○ Fairly healthy
- ○ Very healthy

2. Please tell us if you agree or disagree with the following statements:

	Strongly **disagree**	**Disagree**	Not sure	**Agree**	Strongly **agree**
My child's school actively supports children to eat healthily	○	○	○	○	○
My child's school actively supports children to move more	○	○	○	○	○
It is easy for my children to eat	○	○	○	○	○

	Strongly **disagree**	**Disagree**	Not sure	**Agree**	Strongly **agree**
healthily in this neighbourhood					
It is easy for my children to be physically active in this neighbourhood	◯	◯	◯	◯	◯

Section 2 – About Go Golborne

3. The Go Golborne project has been running since 2015. Please tell us what you think about it below:

	Strongly **disagree**	**Disagree**	Not sure	**Agree**	Strongly **agree**
My family has got some good ideas about healthy eating and moving more from Go Golborne	◯	◯	◯	◯	◯
I don't like Go Golborne telling me what to do	◯	◯	◯	◯	◯
It was easy to make some of the changes that Go Golborne suggests	◯	◯	◯	◯	◯
I've noticed positive changes in my child's school to help children to be healthy	◯	◯	◯	◯	◯
I've noticed positive changes locally that help children to be healthy	◯	◯	◯	◯	◯

3a. Is there anything else you'd like to tell us about the Go Golborne project?

Section 3a – About eating and drinking

Please answer the next questions thinking about your youngest primary school child.

4. How often does your child eat the following for breakfast?

	Never	Less than once a week	1 or 2 days a week	3 or 4 days a week	5 or more days a week
Sugar coated cereals e.g. Frosties, SugarPuffs	○	○	○	○	○
Hi-fibre cereals e.g. Branflakes, Weetabix, Shreddies, Muesli	○	○	○	○	○
Porridge, Ready Brek	○	○	○	○	○
Other cereals e.g. Cornflakes, Rice Krispies	○	○	○	○	

5. Does your child add sugar to their breakfast?

- ○ Yes, always
- ○ Yes, most days
- ○ Sometimes
- ○ Seldom/Not often
- ○ Never

6. How many portions of fruit and vegetables do you think health experts would recommend that a child eats every day?

- 0
- 1
- 2
- 3
- 4
- 5
- 6
- 7 or more
- I don’t know

7. How often does your child do the following?

	Never	Less than once a week	1 or 2 times a week	3 or 4 times a week	5 or 6 times a week	Once per day	2 or more times a day
Drink water with their meals or snacks							
Drink fruit juice or fruit juice drink							
Drink soft drinks such as coke, lemonade, squash (not including diet/sugar free soft drinks)							
Eat chocolate, chocolate bars or sweets							

	Never	Less than once a week	1 or 2 times a week	3 or 4 times a week	5 or 6 times a week	Once per day	2 or more times a day
Eat potato crisps or similar snack							

8. How many portions of fruit and vegetables do you think your child eats each day?

- 0
- 1
- 2
- 3
- 4
- 5
- 6
- 7 or more
- I don’t know

9. Since Go Golborne started in 2015, has your family made any changes to what your child eats/drinks?

	Yes	No
**Any changes to reduce sugar?**		
For example: Swapped to low/no sugar alternatives?		
Cut down on sweets and/or sugary snacks?		
Have smaller portions of sugary foods/drinks?		

	Yes	No
**Any changes to reduce salty/fatty snacks?**	○	○
For example: Swapped salty/fatty snacks for healthier alternatives?	○	○
Smaller portions of salty/fatty snacks?	○	○
Started to check the 'Red/Amber/Green' label on snack packets?	○	○
**Any changes to increase fruit and vegetables?**	○	○
For example: Have more fruit/veg as snacks?	○	○
Add more fruit/veg to our meals?	○	○

10. On most days, how does your child get to and from school?

We are interested in the main transport used, eg: 3 minute walk and 10 minutes by bus = 'public transport'

○ Family vehicle

○ Walk

○ Public transport (bus, train, etc)

○ Bike or scoot

○ Car share

○ Other

11. How far do you live from your child's school?

- Less than 1 mile
- 1-2 miles
- 2-3 miles
- 3-4 miles
- More than 4 miles
- Not sure

12. In a typical week (Monday to Sunday), outside school hours, how often does your child usually exercise or take part in sports activities that makes him or her breathe hard or sweat for at least 20 minutes?
For example: running, skating, swimming laps, tennis, fast cycling, aerobics.

- Never
- 1 or 2 days a week
- 3 or 4 days a week
- 5 or 6 days a week
- Every day

13. Do you know how much physical activity experts recommend for children?
Please indicate below for how often and for how long experts recommend children should do moderate to vigorous physical activity:

How often?

- At least 2 days a week
- At least 4 days a week
- Every day
- I don't know

For how long?

- At least 15 mins

At least 30 mins

At least 1 hour

I don’t know

14. On a typical school day (Monday to Friday), outside school hours, for how long does your child usually do the following?

	Never	Less than 1 hour a day	1 to 2 hours a day	2 to 3 hours a day	3 to 4 hours a day	4 to 5 hours a day	5 or more hours a day
Watch TV, videos or DVDs							
Play on computer, games console, tablet or smart phone							

15. On a typical weekend day (Saturday and Sunday), for how long does your child usually do the following?

	Never	Less than 1 hour a day	1 to 2 hours a day	2 to 3 hours a day	3 to 4 hours a day	4 to 5 hours a day	5 or more hours a day
**Watch TV, videos or DVDs**							
**Play on computer, games console, tablet or smart phone**							

16. Do you know the maximum amount of screen time experts recommend for children?

Screen time includes watching television, playing video games or playing on the computer or on a smart phone.

Half an hour a day 1 hour a day 2 hours a day 4 hours a day I don’t know

○ ○ ○ ○ ○

17. Since Go Golborne started in 2015, has your family made any changes to increase your child's physical activity?

	Yes	No
**Any changes to be more active in travel to/from school?**	○	○
For example: Walk or scoot to/from school more?	○	○
Used more public transport?	○	○
Used the RBKC Walking map?	○	○
**Any changes to increase the amount of active play?**	○	○
For example: Use local parks and adventure playgrounds more often?	○	○
Started going to a new club or sport activity?	○	○
**Any changes to reduce screen time?**	○	○

	Yes	No
For example: Started a time limit for screen use?	○	○
Reduced screen use at meals?	○	○
Tried out some new screen-free Unplug and Play activities?	○	○
Reduced screen use in bedrooms/at bedtimes?	○	○

Q17a. Is there anything else you'd like to say about food, drink or physical activity?

Nearly there! You are moving to the last short section.

Section 4 – information about your youngest child in primary school

Thinking only about your youngest child in primary school:

18. Which primary school does that child go to?

- ○ Ark Brunel
- ○ Barlby
- ○ Bevington
- ○ St Charles
- ○ St Mary's
- ○ St Thomas

19. Which school year is that child in?

- Reception
- Year 1
- Year 2
- Year 3
- Year 4
- Year 5
- Year 6

20. Does your child identify as:

a girl	a boy	Prefer not to say

21. Is your child eligible for benefit-based free school meals?

Yes	No	Don't know	Prefer not to say

22. What is your child's ethnic group?

Please choose one option that best describes your youngest primary school child's ethnic group or background.

White

- English/Welsh/Scottish/Northern Irish/British
- Irish
- Gypsy or Irish Traveller
- Any other White background, please describe:

Mixed/Multiple ethnic groups

- White and Black Caribbean
- White and Black African

- White and Asian
- Any other Mixed/Multiple ethnic background, please describe:

Asian/Asian British

- Indian
- Pakistani
- Bangladeshi
- Chinese
- Any other Asian background, please describe:

Black/ African/Caribbean/Black British

- African
- Caribbean
- Any other Black/African/Caribbean background, please describe:

Other ethnic group

- Arab
- Any other ethnic group, please describe

The end! Thank you very much for your time and answers.
