# Supplementary material for: Impact of a community-based pilot intervention to tackle childhood obesity: a ‘whole-system approach’ case study
Source: BMC Public Health. 2020 Nov 30;20:1818. doi: 10.1186/s12889-020-09694-2 (PMC7708136; doi:10.1186/s12889-020-09694-2)
Supplement: Supplementary file 4 — Additional file 4. The survey distributed to Go-Golborne partners in 2018. (PDF 350 kb) [file 12889_2020_9694_MOESM4_ESM.pdf]

THE ROYAL BOROUGH OF
KENSINGTON
AND CHELSEA

CHSS
University of Kent
Centre for Health Services Studies

A Questionnaire for Go Golborne Partners 2018

First, we would like to ask you some questions about your involvement with Go Golborne.

1. Please tell us how you engaged with the following Go Golborne campaigns.

	5ADAY	Unplug and Play	Sugar Smart	Pedal and Stride
Attended workshops to help plan the campaign	☐	☐	☐	☐
Helped disseminate campaign materials	☐	☐	☐	☐
Participated in the campaign community event	☐	☐	☐	☐
Applied for a grant	☐	☐	☐	☐
Delivered campaign-based activities in your organisation	☐	☐	☐	☐
Attended workshops to help evaluate the campaign	☐	☐	☐	☐
Not applicable to me/my organisation	☐	☐	☐	☐

1a. If you engaged with Go Golborne campaigns in other ways, please tell us what you/your organisation did and for which campaign.

1b. Can you tell us about any impact of this engagement with Go Golborne campaigns on the children and families that you work with?

2. Have you - or, to the best of your knowledge, any colleagues in your organisation - ever attended any training opportunities that have been promoted via the Go Golborne team?

eg: MyTime Active Cooking on a budget or Active Playtime in Schools, Walk Leader training etc.)

- ☐ No
- ☐ Yes. Please tell us what training - and whether it was useful for you/your organisation, with your reasons.

3. Which of the following Go Golborne information and communications have you received or accessed? (please click all that apply)

    - Newsletters
    - Emails and letters from Go Golborne
    - Face to Face or phone contact with the Go Golborne team
    - Go Golborne website
    - Partners area of the Go Golborne website
    - Other. Please tell us what.

4. Have you used or helped to disseminate any of the following resources produced by the Go Golborne team? (please click all that apply)

    - Local information booklets/activity sheets (Things to do, places to go, 45 things to do this summer, Go Golborne activity sheet, 25 ways to Unplug and Play)
    - Go Golborne advertisements (poster, window sticker, postcards)
    - 5ADAY resources (wallchart; family magazine, song/sheet music, poster, fridge magnets, cloth bags)
    - Sugar Smart resources (practical toolkit, window stickers, fridge magnets, top tips leaflets)
    - Family Healthy Weight Care Pathways and Toolkit Walking Time map Snack Recipe cards Various novelty items (water bottles, sports bags, stickers, Frisbees, balls)
    - No, I haven't
    - Other. Please tell us what.

5. Please tell us how you used or helped to disseminate any of the Go Golborne resources produced by the Go Golborne team.

Next, we would like to ask you your thoughts about Go Golborne.

6. Please indicate the extent to which you agree or disagree with the following statements:

	Strongly Disagree	Disagree	Slightly Disagree	Undecided	Slightly agree	Agree	Strongly Agree
'The Go Golborne materials provide useful, relevant information in an easy to read way'	○	○	○	○	○	○	○
'Go Golborne produces information that is highly trustworthy'	○	○	○	○	○	○	○
'It is easy to find the information I want from the Go Golborne website'	○	○	○	○	○	○	○
'I receive regular communication and updates from the Go Golborne team'	○	○	○	○	○	○	○
'There are lots of opportunities for me to get involved with the Go Golborne project'	○	○	○	○	○	○	○

7. Have you made useful contacts as a result of Go Golborne?

- ☐ No
- ☐ Yes. Please provide some examples.

8. Has Go Golborne facilitated opportunities for you to work collaboratively with other organisations?

- ☐ No
- ☐ Yes. Please tell us what sort of opportunities they have facilitated.

9a. How many times have you or your organisation (or partner organisations) applied for a Go Golborne grant?

- ☐ 0
- ☐ 1
- ☐ 2
- ☐ 3
- ☐ 4
- ☐ 5
- ☐ 6 or more

9b. How many times have you been successful?

- ☐ 0
- ☐ 1
- ☐ 2

- 3
- 4
- 5
- 6 or more

10. Has the Go Golborne team provided you with any practical support?

- No
- Yes. Please tell us what sort of practical support they have provided.

11. What did the grant or practical support enable you/your organisation to do? Please note any impact on the children and families that you work with.

12. Since Go Golborne's launch, has your organisation made any of the following changes? (please tick a response for each statement and, where appropriate, use the text boxes to give further detail about how you did this)

	Yes	No	× N/A to our organisation
Introduced at least one measure to promote availability and consumption of water?	○	○	○
Introduced at least one measure to promote availability and consumption of low fat, low sugar snack options?	○	○	○
Introduced at least one measure to enable and encourage active travel?	○	○	○

Introduced at least one measure to enable and encourage active play?	○	○	○
Introduced at least one measure to discourage screen time among children?	○	○	○

12a. Please tell us about any impact that you have noted from these organisational changes on the children and families that you work with.

13. Has Go Golborne inspired you to develop any other work to help improve diet and physical activity in the community since its launch in March 2015? Please provide examples:

14. What factors have contributed to the delivery and impact of Go Golborne in your opinion?

15. Go Golborne was set up as a pilot project. What learning should the team take from this project in your opinion?

16. Has Go Golborne provided any other benefits to you, your organisation or the community not already covered in the questionnaire?

17. Has Go Golborne encouraged you to do anything differently?

- ☐ No
- ☐ Yes. Please tell us how.

18. Has your involvement with Go Golborne inspired you get more involved with your community?

- ☐ No
- ☐ Yes. Please tell us how.

19. Has your involvement with Go Golborne broadened the range of individuals, groups or communities you work with?

- ☐ No
- ☐ Yes. Please tell us how.

Finally we would like to know a bit about you.

24. What is the name of your organisation?

25. Which of the following categories describes your organisation/ group?

- Private Business (for profit)
- Council Department
- VCO (Voluntary Community Organisation)
- Religious organisation or Faith Group School Other (please specify)

26. How many full time equivalent staff work (either paid or unpaid) in your organisation/ group?

- 0
- 1-10
- 11-49
- 50-249
- 250 or more
